# Efficient Photoinduced Electron Transfer from Pyrene‐*o*‐Carborane Heterojunction to Selenoviologen for Enhanced Photocatalytic Hydrogen Evolution and Reduction of Alkynes

**DOI:** 10.1002/advs.202101652

**Published:** 2021-12-26

**Authors:** Xiaodong Yang, Bingjie Zhang, Yujing Gao, Chenjing Liu, Guoping Li, Bin Rao, Dake Chu, Ni Yan, Mingming Zhang, Gang He

**Affiliations:** ^1^ Key Laboratory of Thermo‐Fluid Science and Engineering of Ministry of Education School of Energy and Power Engineering Frontier Institute of Science and Technology Xi'an Jiaotong University Xi'an Shaanxi 710054 P. R. China; ^2^ The First Affiliated Hospital of Xi'an Jiaotong University Xi'an Jiaotong University Xi'an Shaanxi 710054 P. R. China; ^3^ School of Materials Science & Engineering Engineering Research Center of Transportation Materials Ministry of Education Chang'an University Xi'an Shaanxi 710054 P. R. China; ^4^ School of Materials Science and Engineering Xi'an Jiaotong University Xi'an Shaanxi 710054 P. R. China

**Keywords:** *o*‐Carborane, hydrogen evolution reaction, photoinduced electron transfer, pyrene, viologen

## Abstract

A series of pyrene or pyrene‐*o*‐carborane‐appendant selenoviologens (Py‐SeV^2+^, Py‐Cb‐SeV^2+^) for enhanced photocatalytic hydrogen evolution reaction (HER) and reduction of alkynes is reported. The efficient photoinduced electron transfer (PET) from electron‐rich pyrene‐*o*‐carborane heterojunction (Py‐Cb) with intramolecular charge transfer (ICT) characteristic to electron‐deficient selenoviologen (SeV^2+^) (*k*_ET_ = 1.2 × 10^10^ s^−1^) endows the accelerating the generation of selenoviologen radical cation (SeV^+•^) compared with Py‐SeV^2+^ and other derivatives. The electrochromic/electrofluorochromic devices’ (ECD and EFCD) measurements and supramolecular assembly/disassembly processes of SeV^2+^ and cucurbit[8]uril (CB[8]) results show that the PET process can be finely tuned by electrochemical and host–guest chemistry methods. By combination with Pt‐NPs catalyst, the Py‐Cb‐SeV^2+^‐based system shows high‐efficiency visible‐light‐driven HER and highly selective phenylacetylene reduction due to the efficient PET process.

## Graphical abstract

A series of pyrene or pyrene‐*o*‐carborane‐appendant selenoviologens (Py‐SeV^2+^, Py‐Cb‐SeV^2+^) are successfully synthesized. The intramolecular electron transfer (ICT) of Py‐Cb strongly enhances the PET from the donor to electron‐deficient SeV^2+^, which accelerates the formation of selenoviologen radical cations (SeV^+•^) efficiently, enabling enhanced visible‐light‐driven HER and highly selective phenylacetylene reduction.

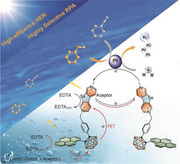

## 1 Introduction

Solar energy is considered a renewable energy source to replace fossil fuels to achieve carbon‐neutral destinations, which could satisfy the growing global energy demand.^[^ ^1^, ^2^ ^]^ The artificial photosynthesis system aims to transfer solar energy to chemical energy and store it in chemical fuels, such as hydrogen, methane or methanol, etc.^[^ ^3^, ^4^, ^5^, ^6^ ^]^ The main steps of artificial photosynthesis involve absorbing photons, converting them into charge‐separated states (electron‐hole pairs) and transferring the electron to a catalyst, where the photocatalytic reaction occurred.^[^ ^7^, ^8^, ^9^ ^]^ It is not difficult to find that an ideal photocatalytic system not only needs an efficient photosensitizer, proper electron mediator and catalyst, but also needs a highly efficient electron transfer process. For the photocatalytic hydrogen evolution reaction (HER), the Pt‐containing compounds or nanoparticles have been proven to be the most efficient catalyst.^[^ ^10^, ^11^ ^]^ Thus, developing novel photosensitizer and electron mediator, as well as accelerating the electron transfer process became the alternative strategy to construction more efficient photocatalytic system.

**Figure 1:**
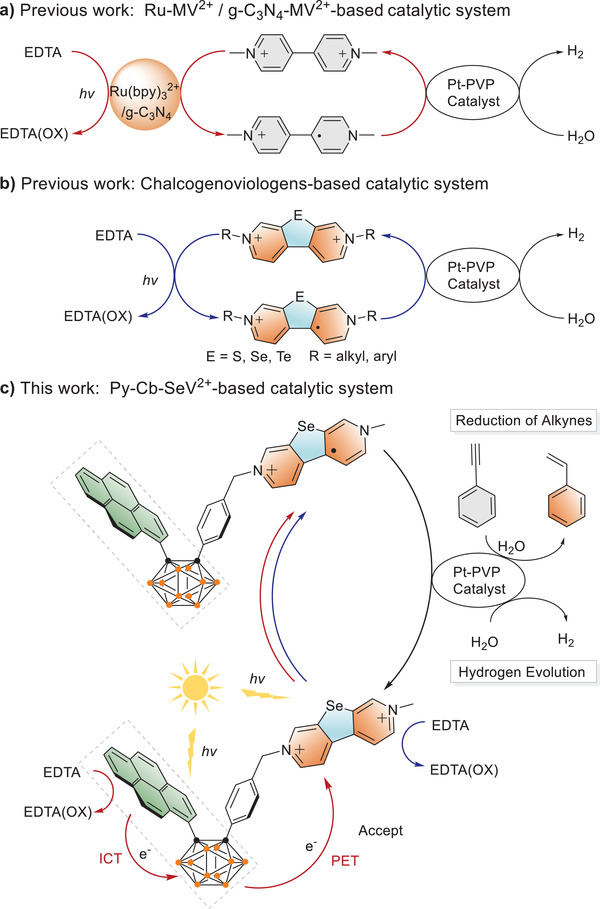
The mechanism of photocatalytic HER and RPA based on viologen‐based systems.

During the past several decades, the photosensitizers have been gained significant progress, lots of photosensitizers, such as nanoparticles,^[^ ^12^, ^13^, ^14^ ^]^ organic dyes^[^ ^15^, ^16^, ^17^ ^]^ and [Ru(bpy)_3_]^2+^ derivatives,^[^ ^18^, ^19^ ^]^ etc. have been reported,^[^ ^20^ ^]^ which showed good light absorption properties in the photocatalytic system. Compared with the photosensitizers, the development of electron mediators received less attention.^[^ ^21^, ^22^, ^23^ ^]^ Among the rarely reported electron mediator, viologen (MV^2+^) was considered as ideal electron transfer agent for photocatalytic reaction due to its multiple redox states and electron accepting properties.^[^ ^19^, ^24^ ^]^ For instance, MV^2+^ can accept electrons from [Ru(bpy)_3_]^2+^ or g‐C_3_N_4_ accompany with the formation of radical cations (MV^+•^) via photoinduced electron transfer (PET) process, followed with HER on Pt‐based catalyst (**Figure** **1**a).^[^ ^25^, ^26^ ^]^ However, the multi‐step electron transfer from photosensitizer to electron mediator to catalyst greatly reduces the utilization of electrons, thereby reducing the efficiency of photocatalytic reactions.

Developing multi‐functional agents which can be used both as photosensitizer and electron mediator provided a new strategy to enhance the photocatalytic performance. However, viologen has a large energy gap (>3.5 eV) due to its noncoplanar spin structure,^[^ ^27^, ^28^, ^29^, ^30^ ^]^ making it unable to be used as a photosensitizer. To solve this problem, our group developed a series of chalcogenoviologens with narrow‐bandgap, fast electron transfer properties, especially selenoviologen (SeV^2+^), which were used as both photosensitizer and electron transfer agent for HER with excellent performance.^[^ ^31^, ^32^ ^]^ Although the design improves and simplifies the photocatalytic system, the production of hydrogen is still far away from the real application due to the slow rate of selenoviologen radical cation (SeV^+•^) formation. Undoubtedly, introducing light trapping group or photosensitizer into SeV^2+^ could accelerate the generation of SeV^+•^ via PET process, and significantly enhance the photocatalytic efficiency.

Different from the conjugated donor small molecule and polymers,^[^ ^33^, ^34^, ^35^, ^36^, ^37^ ^]^ the conjugated D‐A structured heterojunction has been proven to promote electron charge separation within molecules and increase the electron transfer rate.^[^ ^38^, ^39^ ^]^ Recently, using D‐A heterojunction (perylene diimide derivative and poly(fluorene‐*co*‐phenylene)) as electron donor group has successfully realized the biotransformation from carbon dioxide to acetic acid due to the effective separation of electrons and holes and greatly accelerated the electron transfer with bacteria.^[^ ^40^ ^]^ Pyrene (Py) usually acts as a donor due to its good aromaticity and large conjugation system, which has been widely used in the field of organic optoelectronic materials.^[^ ^41^, ^42^, ^43^ ^]^ The efficient electron transfer between pyrene derivatives and viologen has also been widely used in biochemical and medicinal applications.^[^ ^44^, ^45^ ^]^ The pyrene‐*o*‐carborane (Py‐Cb) heterojunction composed with Py and typical electron‐deficient *o*‐carborane has shown excellent electron transfer characteristics, charge separation, and optical properties as a result of the intramolecular charge transfer (ICT) process.^[^ ^46^, ^47^, ^48^, ^49^ ^]^ It can be envisioned that introducing the Py or Py‐Cb units as donors to transfer the electrons to electron‐deficient SeV^2+^ should efficiently accelerate the generation of SeV^+•^ via PET process, which could significantly improve the photocatalytic performances of HER and organic reactions.

Based on the considerations, pyrene or pyrene‐*o*‐carborane‐appendant selenoviologens (Py‐SeV^2+^, Py‐Cb‐SeV^2+^) and viologens (Py‐MV^2+^, Py‐Cb‐MV^2+^ for comparison) were synthesized by using Py or Py‐Cb dyad as donor groups and SeV^2+^ or MV^2+^ as acceptors. The efficient ICT of Py‐Cb heterojunction enhanced the PET from Py‐Cb to SeV^2+^ in Py‐Cb‐SeV^2+^, of which electron transfer rate is the highest than other analogues, accelerating the generation of selenoviologen radical cation (SeV^+•^). The controllable PET process can be finely tuned by the spectroelectrochemistry and host–guest chemistry methods. By using Pt nanoparticles as the catalyst, the Py‐Cb‐SeV^2+^ system showed higher photocatalytic efficiency in HER and realized the reduction of phenylacetylene reaction (Figure 1).

## 2 Results and Discussion

**Scheme 1:**
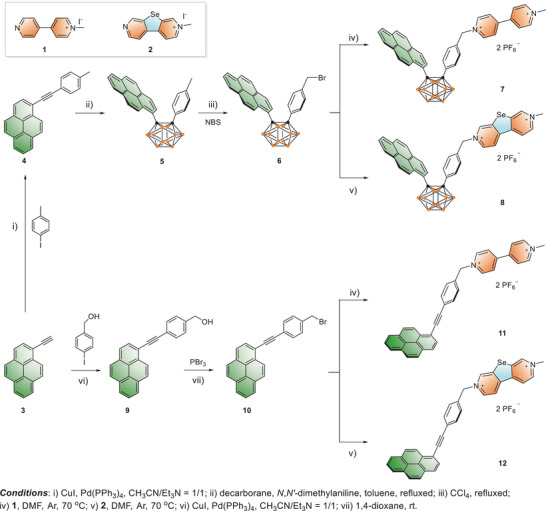
Synthetic approach toward Py‐MV^2+^ (**7**), Py‐Cb‐SeV^2+^ (**8**), Py‐MV^2+^ (**11**), and Py‐Cb‐SeV^2+^ (**12**).

The synthetic route for pyrene or pyrene‐*o*‐carborane‐appendant selenoviologens (Py‐SeV^2+^, Py‐Cb‐SeV^2+^) were shown in **Scheme** **1**. First, **4** was synthesized from 1‐ethynylpyrene (**3**)^[^ ^46^ ^]^ and *p*‐iodotoluene through Sonogashira coupling reaction with PdCl_2_(PPh_3_)_2_ and CuI as cocatalyst. Then toluene‐4‐*o*‐carborane‐pyrene (**5**) was obtained by insertion reaction of **4** into B_10_H_10_ with 45% yield. Compound **6** was synthesized through bromomethyl reaction from **5** and NBS in CCl_4_ solution. Py‐Cb‐SeV^2+^ (**8**) was obtained by ionization **2** with **6** in DMF solution at 70 °C for 2d with 51% yield. Alternatively, (4‐(pyren‐1‐ylethynyl)‐phenyl)methanol (**9**) was obtained from **3** and (4‐iodophenyl)methanol under a similar condition with the formation of **4**. Compound **10** was synthesized via electrophilic substitution reaction from PBr_3_ and **9**. Similarly, compound **12** was obtained with an 85% yield. For comparison, the pyrene and pyrene‐*o*‐carborane‐appendant methyl viologens (Py‐MV^2+^, **11**; Py‐Cb‐MV^2+^, **7**) were also synthesized by using monomethylated bipyridine (**1**) as viologen source (Supporting Information).

**Figure 2:**
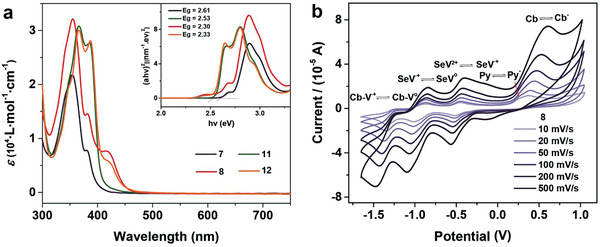
a) The UV–vis spectra of **7**, **8**, **11**, and **12** in DMF, [M] = 5 × 10^−4^ m; extrapolated optical bandgaps (*E*_g_) are shown as insets. b) Cyclic voltammograms of **8** in DMF solution, [*n*‐Bu_4_N][PF_6_] (0.1 m) as supporting electrolyte, versus Fc/Fc^+^, [**8**] = 1 × 10^−3^ m.

To elucidate the electronic structures of the compounds, the optical and electrochemical spectral of as‐prepared compounds were characterized by UV−vis and cyclic voltammetry (CV) measurements. The UV−vis absorption spectra were shown in **Figures** **2**a and Figures S1 and S2 (Supporting Information), and their photophysical datum were depicted in Table S1 (Supporting Information). As shown in Figures S1 (Supporting Information), the maximum absorptions of **1**, **2**, **6**, and **9** are 280 nm, 398 nm, 375 nm, 395 nm in the DMF solution, respectively. Compared with monomethylated viologens (**1** and **2**) and brominated compounds (**6** and **9**), the maximum absorptions of viologen derivatives (**7**, **8**, **11**, and **12**) significantly red‐shifted to around 419 nm may due to the molecular orbital hybridization and PET process between electron donors (Py and Py‐Cb) and acceptors (MV^2+^ and SeV^2+^).^[^ ^50^ ^]^ Furthermore, their maximum absorption peaks are slightly influenced by solvent polarity (Figure S2, Supporting Information), which can be attributed to the CT of molecules.^[^ ^51^ ^]^ Besides, the theoretical maxima of the absorption spectra of **7**, **8**, **11**, and **12** peaks at 365, 355, 361, and 360 nm, respectively, which were consistent with the experimental data (Figures S21–S24, Supporting Information). According to the photophysical data, the calculated optical bandgaps of **7**, **8**, **11**, and **12** are 2.61, 2.30, 2.53, and 2.33 eV, respectively, which means that the four viologen substitutes are excellent semiconductor materials suitable for photocatalytic reactions.^[^ ^52^, ^53^ ^]^ Furthermore, the first singlet excitation energy of the four viologens were obtained by time‐dependent DFT (TD‐DFT) calculations (at the TD‐PBE0/6‐31G*//PBE0/6‐31G* level of theory), the results have shown that the molecular orbital density for HOMO and LUMO were delocalized (Figure S25 (Supporting Information)), which indicated that the electron and the hole in molecules were easy to separate after absorption a photon of light and extend the life time of electrons.^[^ ^54^, ^55^ ^]^ As shown in Figure S26 (Supporting Information), the HOMO, LUMO energy levels are almost the same, which indicated that the Py‐Cb unit and pyrene have similar electronic properties. In addition, the LUMO energy levels of **6** is higher than **2**. Therefore, Py‐Cb can be acted as an electron donor, and SeV^2+^ is an electron acceptor in Py‐Cb‐SeV^2+^.

The CV spectra under different scan rates were tested as shown in Figure 2b and Figure S3 (Supporting Information). The results suggested that the four viologen derivatives have good electrochemical reversibility. In detail, **8** presents redox potentials *E*_red1,1/3_ = −0.58 V (*E*_LUMO_ = −4.22 eV), *E*_red2,1/3_ = −1.04 V, *E*_red3,1/3_ = −1.42 V, and *E*_ox1,1/2_ = 0.14 V, *E*_ox2,1/2_ = 0.46 V. Compared with **5** (*E*_ox_ = 0.46 V) and **9** (*E*_ox_ = 0.15 V), the two‐oxidation peaks of **8** at around 0.14 and 0.45 V may be attributed to pyrene and *o*‐carborane unit, respectively. Besides, **7** and **8** have an extra reduction potential at around −1.4 V than **11** and **12** due to molecular hybridization, which are consistent with previous work about carborane‐based viologen materials.^[^ ^56^ ^]^ The molecular HOMO and LUMO values versus NHE were calculated based on UV–vis and CV data (Figure S14, Supporting Information), which indicated that compounds **7**, **8**, **11**, and **12** may provide enough thermodynamic driving force for HER.

**Figure 3:**
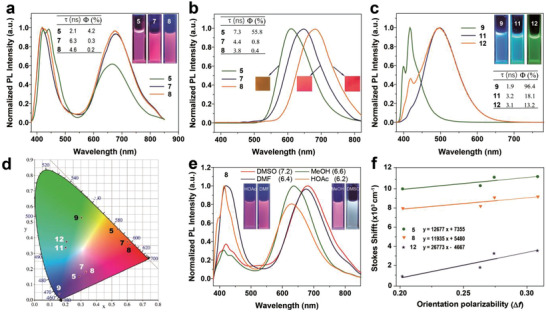
Fluorescence spectra of a) **5**, **7**, and **8** in DMF (5 × 10^−5^ m). b) **5**, **7**, and **8** in solid state. c) **9**, **11**, and **12** in DMF (5 × 10^−5^ m). d) CIE chromaticity of **5**, **7**–**9**, and **11**–**12** in DMF solution (white word) and in solid state (black word). e) Fluorescence spectra of 8 in different solvent (5 × 10^−5^ m). f) Lippert–Mataga plot for **5**, **8**, and **12** in different solvents.

To understand the relationships between the molecular structures and optical properties, the emission properties of the molecules in both solution and solid states were tested. As shown in **Figure** **3**a, **5**, **7**, and **8** show the similar dual emissions accompanying with weak fluorescence in DMF. The emissions around 430 nm was originated from the LE states of the pyrene units, and the broad emissions around 680 nm are attributed to the CT emissions from the Cb‐Py heterojunction to MV^2+^ or SeV^2+^.^[^ ^57^ ^]^ Compared with **5**, the maximum emission bands of **7** and **8** are red‐shifted from 600 to 650 and 700 nm in the solid state (Figure 3b), and the fluorescence quantum yields (Φ) of **7** and **8** obviously decreased from 55.8% to around 0.7% and 0.4%, respectively. The redshifted emission and fluorescence quenching phenomenon confirmed the PET process and the ICT between Cb and Py moiety can enhance the PET process. In contrast with **7** and **8**, **11** and **12** still show strong fluorescence at around 510 nm with Φ = 18.1%, 13.2%, respectively, which suggested that PET process in **7** and **8** is more efficient than **11** and **12** (Figure 3c). Based on the fluorescent data, the CIE chromaticity coordinates were prepared, which were almost the same as the actual luminescence of molecules (Figure 3d).

To further explore the electron transfer within the molecule, the electron‐transfer constants (*K*_ET_) were calculated according to the molecular photoluminescence lifetimes (*τ*) and quantum yield. The results were summarized in Table S2 (Supporting Information). The *K*_ET_ of **7**/**8** (7.22 × 10^9^ for **7**; 1.20 × 10^10^ for **8**) were an order of magnitude more than **11**/**12** (2.29 × 10^9^ for **11**; 3.34 × 10^10^ for **12**), which were attributed to the carborane moiety enhancing the electron transfer rate. Although the *K*_ET_ of carborane‐based viologen has not been reported, the *K*_ET,_ **_11_** and *K*_ET,_ **_12_** are basically consistent with the literatures.^[^ ^58^ ^]^ In order to further verify this result, the electrical conductivities of **7**, **8**, **11**, and **12** were tested. The results show that the electrical conductivities of **7**, **8**, **11**, and **12** are 1.10 × 10^−8^, 9.93 × 10^−9^, 1.16 × 10^−9^ and 8.7 × 10^−10^ S m^−1^, respectively, which indicate that the introduction of carborane into molecules (**7** and **8**) can effectively improve electron transfer.^[^ ^56^ ^]^ Thus, **7** and **8** might exhibit better photocatalytic performance due to the faster electron transfer rate. Besides, the emission spectra were studied in different solvents with different polarities such as DMSO (7.2), MeOH (6.6), DMF (6.4), and AcOH (6.2), which were shown in Figures 3e and S5 (Supporting Information). It can be seen that all the compounds showed dual emission bands at around 430 and 650 nm, and the emission band around 430 nm was barely affected, but emission band around 650 nm was blue shifted by increasing the solvent polarity. The solvent polarity dependence of the CT emission was further investigated using the Lippert–Mataga relationship. A significant linear relationship was obtained from the plots with the peak positions observed around 650 and 520 nm, which can be indicative of an ICT state (Figure 3f). In addition, the transition dipole moment was estimated as 5.10 D (**5**), 9.29 D (**8**), and 18.71 D (**12**) according to the slope of the Lippert–Mataga plot and the Onsager radius of 5.91 Å (**5**), 8.99 Å (**8**), and 10.95 Å (**12**). These values are relatively larger than those of previous luminescent dyes with ICT emission properties,^[^ ^58^ ^]^ which suggested that structural alteration may occur in the excited‐state. Based on the UV–vis, fluorescence and electrochemical data from the preceding paragraph, we are in a position to estimate the driving force for electron transfer between electron donors (Py and Py‐Cb) and acceptor in the four compounds shown in **Table** **1**. The resulsts show that the ΔG^0^ of molecules **7**, **8**, **11**, **12** are less than zero, indicating that there is a PET process in the molecule, which is consistent with the reported results in the literatures.^[^ ^59^, ^60^ ^]^

**Table 1:** Calculated driving force (Δ*G*°) for **7**, **8**, **11**, **12** in MeOH and DMSO at 25 °C

Compound	*E*_OX_ [V]^a)^	*E*_red_ [V]^b)^	*E*_00_ [eV]^c)^	Δ*G* ^0^ [eV]^d)^	
				MeOH	DMSO
**7**	0.45	−0.46	1.89	−1.06	−0.90
**8**	0.46	−0.58	1.89	−0.93	−0.77
**11**	0.15	−0.64	2.74	−2.01	−1.89
**12**	0.21	−0.62	2.74	−1.97	−1.85

^a,b)^The donor and acceptor redox potentials in DMF;

^c)^ determined from absorption and fluorescence measurements;

^d)^ calculated from $\Delta G^{0}=\;e\left(E_{\mathrm{OX}}-E_{\mathrm{red}}\right)-\;E_{00\;}+\;\frac{e^{2}}{4\pi \varepsilon_{0}}\left(\frac{1}{\varepsilon_{s}}-\;\frac{1}{\varepsilon_{s}^{\mathrm{ref}}}\right)\left(\frac{1}{r}\right)$.

**Figure 4:**
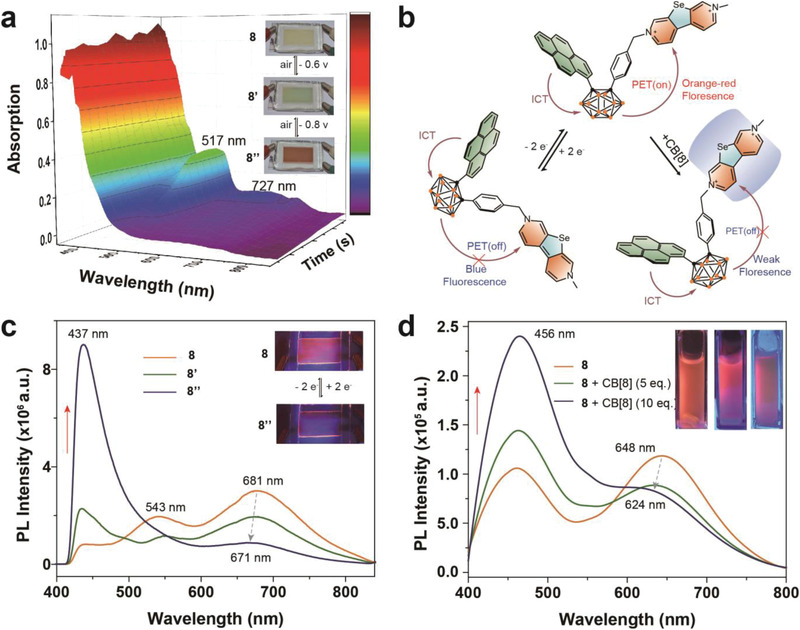
a) Spectroelectrochemistry of **8** and images of ECD color changes for three are shown as the inset. b) Electrochemical and supramolecular chemistry methods to control the intramolecular PET process. c) Fluorescence spectra of **8** in DMF solution and images of EFCD color changes for three states are shown as the inset. d) Fluorescence spectra of **8** without and with CB[8] in H_2_O and images of color changes are shown as the inset.

To better understand the PET process, the UV−vis and fluorescence spectroelectrochemistry based on electrochromic and electrofluorochromic devices (ECD and EFCD) were studied in situ. Take **8** as an example, the DMF solution of **8** as an active component was injected into the cavity, which was made up of two state indium‐tin oxide (ITO)‐coated glasses and double side adhesive tape (Figure S6, Supporting Information). When −0.6 V voltage was applied, the color of the device window changed from light yellow to green with the absorption bands at 517, 651, and 728 nm increasing due to the formation of the radical cation (**8’**). Along with the voltage being further increased to ‐0.8 V, the maximum absorption peak gradually decreased with red color presentation, which may be attributed to the formation of neutral state (**8’’**) (**Figure** **4**a). The results were consistent with the chemical reduction of Zn or Na (Figure S9, Supporting Information). To further prove the existence of two states (**8’** and **8’’**), the EPR spectroscopy was tested. The results show that the g‐factor is 2.0023 for Zn reduction and no signal for Na reduction, which are consistent with the previous work (Figure S11, Supporting Information).^[^ ^31^ ^]^ In addition, the emission at 681 nm was decreasing along with the −0.6 V voltage applied, and the emission band at 437 nm was increasing until the voltage up to −0.8 V. Then the emission peak was slightly blue shifted to 671 nm (Figure 4c). This data confirmed that the PET in the molecules was turned off after the SeV^2+^ unit getting two electrons to neutral state. Meanwhile, **7**, **11**, and **12** showed similar phenomena (Figures S7–S10, Supporting Information).

To further insight into electron transfer dynamics between donor (Py‐Cb heterojunction) and acceptor (SeV^2+^), we established a supramolecular model combination of a host (cucurbit[8]uril, CB[8]) and guest (SeV^2+^).^[^ ^61^, ^62^, ^63^ ^]^ When added CB[8] (2 × 10^−3^ m) to **8** (5 × 10^−6^ m, 2.5 mL in water), the results presented a significant decrease of the emission band at around 648 nm, which was accompanied by a wide peak around 436 nm. When CB[8] was dropped to ten equivalents of **8**, the maximum emission peak was obviously blueshifted to 624 nm, and the emission peak at 460 nm had a significant increase (Figure 4c). The results suggested that CB[8] also could prevent the electron transfer process, which were consistent with the previous result.^[^ ^50^ ^]^ Clearly, both electrochemistry and supramolecular chemistry can efficiently regulate the intramolecular PET process (Figure 4b).

**Figure 5:**
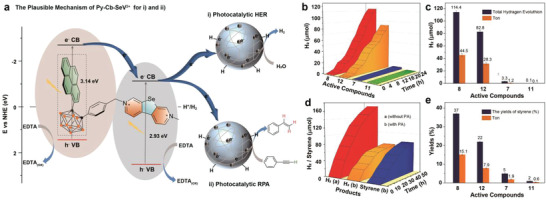
a) The plausible mechanism of Py‐Cb‐SeV^2+^ for photoinduced hydrogen evolution and reduction of phenylacetylene (RPA). b) Time‐dependent hydrogen generation from aqueous solution under xenon lamp. c) Total hydrogen generation of active components. d) Hydrogen evolution and styrene formation versus reaction times. e) The yields of styrene versus active components.

Considering the strong visible‐light absorption, narrow bandgaps, efficient charge separation and fast electron transfer rate of Py‐Cb‐SeV^2+^, a visible‐light photocatalyst hydrogen evolution system was designed. Taking **7**, **8**, **11**, and **12** as active compounds, colloidal platinum particles (Pt‐PVP) as catalysts, and EDTA as electron sacrifice agent, the reaction was taken place in a pyrex bottle (20 mL) with buffer solution (pH = 5.0, 5 mL) under Xenon light (*λ* > 400 nm) at 100 mW. As the results are shown in **Figure** **5**b,c, hydrogen evolution of **7** (3.3 µmol) significantly increased after irradiation 24 h compared with **11** (total hydrogen evolution 0.1 µmol), which could be attributed to the ICT process can enhance the PET efficiency of the molecule (Figure S19, Supporting Information).^[^ ^64^ ^]^ Interestingly, the total hydrogen evolution of **12** (34.4 µmol total 12 h) is about 3 times more than Se‐BnV^2+^(13.04 µmol total 12 h),^[^ ^32^ ^]^ which indicated that the PET process greatly improved the molecular absorption and significantly accelerate the generation of SeV^+•^, resulting in the improved efficiency of HER (Figure S20, Supporting Information). Compared with **7** and **11**, the hydrogen evolution of **8** and **12** significantly increased. Benefit from the special molecular structure, **8** generated 114.4 µmol hydrogen (TON: 44.5, hydrogen generation rate: 1.91 mmol h^−1^ g^−1^, apparent quantum yield AQY: 0.79%), which is much higher than **12** (82.8 µmol hydrogen, TON: 28.3, hydrogen generation rate: 1.34 mmol h^−1^ g^−1^, AQY: 0.54%), due to the more efficient generation of SeV^+•^. Considering the dispersibility of the catalyst in water has a great influence on the photocatalytic effect, the dispersibility of the PCs was studied by TEM spectra. As shown in Figure S12 (Supporting Information), **7** and **8** are better dispersed in water than **11** and **12** due to the hydrophobic nature of carborane unit. Furthermore, almost no hydrogen was detected without PCs or irradiation in **8**‐based system, which means the catalytic system is a complete photocatalytic system. When the molecules **4**, **5**, and **9** were physically mixed with selenoviologen or viologen as a catalyst to participate in photocatalytic hydrogen evolution, the experimental results show that simple physical mixing does not significantly improve the hydrogen production effect of the viologen system, which further shows that the PET strategy is effective (Table S3, Supporting Information). To further clarify the stability of the molecules in the process of photocatalytic hydrogen evolution, molecule **8** with the best catalytic performance was selected to test cycle stability, and every 12 h is a cycle. The experimental results showed that the hydrogen production effect could still be maintained at about 82% after five cycles (Figure S27, Supporting Information). After 60 h light irradiation, it is not difficult to find from the ^1^H NMR data of molecule **8** that more than 80% of molecule **8** remains unchanged, which indicate that the molecules have good light stability (Figure S28, Supporting Information). To the best of our knowledge, the hydrogen production efficiency of **8** is the highest value compared with other organic viologen‐containing photocatalytic hydrogen evolution systems (**Table** **2**).

**Table 2:** Hydrogen generation activities of some organic photocatalytic system

Classify	Catalysts	Hydrogen generation rate [mmol h^−1^ g^−1^]	TON/AQY	Conditions	Reference
Metal‐bearing organic small molecule	MV(NO_3_)_2_ {[Ru(bpy)_3_] (NO_3_)_2_•3H_2_O, Co(dmgH)_2_(pyridine)Cl}	0.016	–	BS.^a)^	[70]
	PBDT‐BPY (10−20% CoCl_2_)	0.14	0.025% (AQY)	30 vol% diethylamine/water	[71]
	[PtCl_2_(dpbpy MV_4_)]Cl_8_ •16H_2_O	0.33	—	BS.	[72]
	Zn(II)PPIX/C_3_N_4_ hybrid system; MV^2+^ (PT)	5.67	2.2 (TON)	Tris‐buffer	[73]
Organic small molecule with photosensit izer	MV^2+^ (PT)	0.074	0.064% (AQY)	Pure aqueous *λ* > 420 nm	[33]
	SWCNT/ dendrimer nanohybrids; MV^2+^ (PT)	0.074	—	Tris‐HCl buffer	[74]
	Thiocarbonyl dye/SWCNT/C60; MV^2+^ (PT)	0.098	7.6 (TON)	Tris‐HCl buffer	[75]
Organic small molecule without metal	Se‐BnV^2+^	0.31	0.17% (AQY)	Pure aqueous *λ* > 400 nm	[32]
	Se‐PhV	0.71	11.4 (TON)	BS., *λ* > 400 nm	[31]
	**8**	1.91	44.5 (TON) 0.79% (AQY)	BS., *λ* > 400 nm	This work.
	**12**	1.34	28.3 (TON) 0.54% (AQY)	BS., *λ* > 400 nm	This work.

^a)^ Aqueous acetate buffer solution (BS., 0.03 m CH_3_COOH and 0.07 m CH_3_COONa).

Similar to photocatalytic HER, photocatalytic organic reactions are also considered to be an effective way to solve the energy crisis. Alkene is an important building block in various natural products, pharmaceuticals and agrochemicals. It was usually obtained via selective hydrogenation of alkynes.^[^ ^65^, ^66^ ^]^ However, it is still a challenge to obtain both high conversion and selectivity. Therefore, we choose Py‐Cb‐SeV^2+^ to study the photocatalytic reduction of phenylacetylene to styrene. Based on the optimal photocatalytic hydrogen production conditions, an additional organic solvent (DMF, CH_3_CN, DCM, MeOH, cyclohexane) was added to promote the dissolution of phenylacetylene in the system. After 48 h of irradiation, styrene was only detected in the system containing cyclohexane (5% yields). When the ratio of cyclohexane/buffer solution was reduced from 4/1 to 1/1, the yield of styrene was significantly increased to 24%. When the amount of additive was increased 2.5 times, the desired product styrene was obtained in higher yields (37%, Table S4, entry 10, Supporting Information). After screening the acid strength of the buffer solution, the results showed that weak acidity (pH = 5.0) was beneficial to product formation. Under the optimal conditions, hydrogen evolution and styrene formation versus reaction times were studied. As shown in Figure 5d, it is beneficial to generation hydrogen at the beginning of the reaction. When the concentration of hydrogen in the system reaches a certain value (3.2 µmol mL^−1^), the production rate of styrene begins to be faster than hydrogen. For comparison, the system generated 172 µmol hydrogen within 48 h without phenylacetylene. These results showed that the production of styrene and hydrogen was always in a competitive relationship. Other active compounds (**7**, **11**, and **12**) were also tested in this reaction. Among them, **8** showed the best performance with yields of 37% and TON of 15.1. Importantly, almost no reduction products of phenylethane were detected (Figure 5e). In order to further expand the scope of application of photocatalytic reduction of alkynes, the low toxicity of alkyl alkyne (1‐hexyne) has also been investigated. After photocatalytic reduction, the target product 1‐hexene can be obtained with a yield of 21% and a selection of more than 99%. The high selectivity may be attributed to the competitive hydrogen production and alkyne reduction, preventing the formation of a completely reduced byproduct, alkane.

To further understand the photocatalytic reaction mechanism, the supramolecular method was employed by using CB[8] and amantadine (AD) through supramolecular assembly and disassembly processes.^[^ ^67^, ^68^, ^69^ ^]^ Take **8** as an example, the total hydrogen evolution is 16.3 µmol after irradiation 6 h, and the total hydrogen evolution markedly decreased to 2.7 µmol with the CB[8] being added for irradiation 6 h. Once AD was added and irradiated another 6 h, the hydrogen evolution increased to 11.6 µmol. The results indicated that the CB[8] may combine with SeV^2+^ to inhibit electron transfer and AD resolve the SeV^2+^ to reproduce hydrogen (Figure S15, Supporting Information). Besides, the UV–vis spectrum of **8** and **8**+CB[8] were tested under different light periods. The results showed that the generation of radical cations was not affected by CB[8] (Figure S16, Supporting Information). The ^1^H NMR of **8**, **8**+CB[8] and **8**+CB[8]+AD in the D_2_O with 100 × 10^−3^ m NaCl were tested respectively. According to the ^1^H NMR spectra, the signals for the protons on pyridyl groups shifted to up‐field, and the signals for the protons on methyl and methylene shifted low field, which indicated that the pyridyl groups were encapsulated into the cavity of CB[8], methyl and methylene were outside of CB[8] (Figure S17, Supporting Information), which confirm the host–gust interactions. All the experimental and theoretical results confirmed the photocatalytic mechanism: under visible light, SeV^2+^ unit can absorb photons to generate SeV^+•^. Meanwhile, excited Cb‐Py heterojunction can transfer electrons to SeV^2+^ unit via the PET process to accelerate the generation of SeV^+•^. After the electrons transferred from SeV^+•^ to Pt‐PVP catalyst, the HER or reduction reaction occurred on the surface of the Pt catalyst (Figures 1 and 5a).

## 3 Conclusion

A series of pyrene or pyrene‐*o*‐carborane‐appendant selenoviologens (Py‐SeV^2+^, Py‐Cb‐SeV^2+^) was successfully synthesized. The intramolecular electron transfer (IET) of Py‐Cb heterojunction strongly enhance the PET from the donor to electron‐deficient SeV^2+^ compared with the Py‐SeV^2+^, Py‐MV^2+^, and Py‐Cb‐MV^2+^, which accelerate the formation of selenoviologen radical cations (SeV^+•^) efficiently. The Py‐MV^2+^ and Py‐Cb‐MV^2+^ showed ACQ effect, and the Py‐SeV^2+^, Py‐Cb‐SeV^2+^ showed AIE property due to the CT process. It was found that the PET process can be controlled by the electrochemical or host–guest chemistry methods via electrochromic and electrofluorochromic devices’ (ECD and EFCD) measurements and supramolecular assembly/disassembly processes of SeV^2+^ and cucurbit[8]uril (CB[8]). Py‐Cb‐SeV^2+^ were used as electron mediator and photosensitizer for controllable HER and selectively reduction of phenylacetylene to styrene due to the excellent optoelectronic properties. Notably, the Py‐Cb‐SeV^2+^ showed the highest reported value to date for viologen‐based visible‐light‐driven HER. This contribution provided a new strategy to develop highly efficient viologen analogues‐based organic photocatalyst, which laid a solid foundation for the solar energy conversion.

## Conflict of Interest

The authors declare no conflict of interest.

## Supporting information

Supporting Information

## Data Availability

The data that support the findings of this study are available in the supplementary material of this article and are available from the corresponding author upon reasonable request.
