# Supplementary material for: Efficient Photoinduced Electron Transfer from Pyrene‐o‐Carborane Heterojunction to Selenoviologen for Enhanced Photocatalytic Hydrogen Evolution and Reduction of Alkynes
Source: Adv Sci (Weinh). 2021 Dec 26;9(5):2101652. doi: 10.1002/advs.202101652 (PMC8844576; doi:10.1002/advs.202101652)
Supplement: Supplementary file 1 — Supporting Information [file ADVS-9-2101652-s001.pdf]

ADVANCED SCIENCE
Open Access

Supporting Information

for *Adv. Sci.,* DOI: 10.1002/advs.202101652

Efficient Photoinduced Electron Transfer from Pyrene-o-Carborane Heterojunction to Selenoviologen for Enhanced Photocatalytic Hydrogen Evolution and Reduction of Alkynes

Xiaodong Yang, Bingjie Zhang, Yujing Gao, Chenjing Liu, Guoping Li, Bin Rao, Dake Chu, Ni Yan, Mingming Zhang and Gang He**

Supporting Information

Efficient Photoinduced Electron Transfer from Pyrene-o-Carborane Heterojunction to Selenoviologen for Enhanced Photocatalytic Hydrogen Evolution and Reduction of Alkynes

Xiaodong Yang, Bingjie Zhang, Yujing Gao, Chenjing Liu, Guoping Li, Bin Rao, Dake Chu, Ni Yan, Mingming Zhang and Gang He**

Experimental Procedures

1. Materials and instrumentation

General. All reactions were performed using standard Schlenk and glovebox (Vigor) techniques under argon atmosphere. toluene, THF were distilled from sodium/benzophenone prior to use. Extra dry solvents (CH_3CN, DMF,1,4-dioxane, carbon tetrachloride, Et_3N), 1-bromopyrene (98%), $Pd(PPh_3)_2Cl_2$ (99%), CuI (99%), Ethynyltrimethylsilane (99%), TBAF (1.0 mol/L in THF), $Pd(PPh_3)_4$ (99%), *N*,*N*-dimethylaniline (99%), azobisisobutyro nitrile (AIBN) (99%), *N*-bromosuccinimide (NBS) (99%) and PBr_3 (99%) were purchased from Energy Chemical Inc. Decaborane (99%) was purchased from Zhengzhou Alfa Chemical Co. Ltd. trimethyl(pyren-1-ylethynyl)silane [1] was prepared according to literature procedures, if no other special indicated, other reagents and solvents were used as commercially available without further purification. Column chromatographic purification of products was accomplished using 200-300 mesh silica gel.

NMR spectra were measured on a Bruker Avance-400 spectrometer in the solvents indicated; chemical shifts are reported in units (ppm) by assigning TMS resonance in the ^1H spectrum as 7.26 ppm, $CDCl_3$ or 2.50 ppm, DMSO-d^6 resonance in the ^{13}C spectrum as 77.0 ppm or 35.0 ppm. Coupling constants are reported in Hz with multiplicities denoted as s (singlet), d (doublet), t (triplet), q (quartet) and m (multiplet). UV-vis measurements were performed using DH-2000-BAL Scan spectrophotometer. Fluorescence measurements were conducted on an FLS920 system and FLS980 system. The cyclic voltammetry (CV) was measured using CHI660E, with a polished gold electrode as the working electrode, a Pt-net as counter electrode, and an Ag wire as reference electrode, using ferrocene/ferrocenium (Fc/Fc^+) as internal standard. High-resolution mass spectra (HRMS) were collected on a Bruker maXisUHR-TOF mass spectrometer in an ESI positive mode. Analytical gas chromatography (GC) for gas sample were carried out on an agilent (8860, N_2 carrier gas). EPR was measured using a Bruker EMX PLUS6/1 instrument at room temperature in dry degassed DMF. The

300 W xenon lamp used for irradiation and the optical power meter were supplied by Beijing Perfect Light Co. Ltd. Photographs were taken using a Nikon D5100 digital camera.

All the computational calculations reported in this work were performed using the Gaussian 09 code. [2] To simulate the experimental UV-Vis in *N*,*N*-Dimethylformamide (DMF), the Polarizable Continuum Model (PCM) as a self-consistent reaction field (SCRF) was used for the calculation of equilibrium geometries, vibrational frequencies and excited state calculations. The geometries for the ground state of these compounds in the DMF solution were optimized at TD-PBE0/6-31G* //PBE0/6-31G* [3] basis and the keyword “opt=tight” was used. All isosurface values are ±0.03.

The Lippert-Mataga equation is as follow:

$$\Delta \nu = \nu_{\mathrm{abs}} - V_{em} = \frac{2\Delta f}{\mathrm{h}ca^3}(\mu_e - \mu_g)^2 + b \quad (1)$$

$$\Delta f = \frac{\varepsilon - 1}{2\varepsilon + 1} - \frac{n^2 - 1}{2n^2 + 1} \quad (2)$$

in which $\Delta\nu = \nu_{abs}$ - ν_{em} stands for Stokes shift, ν_{abs} and ν_{em} are absorption and emission frequency (cm^{-1}), *h* is the Planc’s constant, *c* is the velocity of light in vacuum, a is the Onsager radius and b is a constant. Δf is the orientation polarizability, ε is the refractive index, n is the dielectric constant, μ_e and μ_g are the dipole moments of the emissive and ground states, respectively. $(\mu_e\text{-}\mu_g)^2$ is proportional to the slope of the Lippert-Mataga plot.

The ΔG^0 equation is as follow:

$$\Delta \mathrm{G}^0 = e(E_{OX} - E_{red}) - E_{00} + \frac{e^2}{4\pi\epsilon_0}\left(\frac{1}{\epsilon_s} - \frac{1}{\varepsilon_s^{ref}}\right)\left(\frac{1}{r}\right) \quad (3)$$

in which the donor and acceptor redox potentials (E_{ox} and E_{red}), the E_{00} excitation energy of the donor is the energy approximated with the cross point of absorption wavelength and fluorescence wavelength, and the average radii of the donor and acceptor (r). dielectric constant of the given solvent (MeOH, 32.6; DMSO, 48.9). $\varepsilon_{\mathrm{ref}}$ is the dielectric constant of the reference solvent used in electrochemistry (DMF: 37.6).

Apparent quantum yield (AQY) calculation:

For the max of the absorption of **8** is 419 nm, in the equation (1), λ = 419 nm. E is measured 100 mW. The number of incident photons (N_0) is calculated to be 2.11×10^{17} s^{-1} by equation (1). The H_2 molecules generated in 24 h under Xenon light was 114.4 μmol. The photons number of collected to be H_2 is 7.97×10^{14} s^{-1} as calculated by equation (2). The AQY is 7.55×10^{-3} calculated by equation (3):

$N_0 = \lambda E/hc = 419 \times 10^{-9} \times 100 \times 10^{-3} / (6.63 \times 10^{-34} \times 3 \times 10^{8})\ s^{-1} = 2.11 \times 10^{17}\ s^{-1}$ (1)

$N = nNA/t = 114.4 \times 10^{-6} \times 6.02 \times 10^{23} / 24 \times 3600\ s^{-1} = 7.97 \times 10^{14}\ s^{-1}$ (2)

$AQY_{\mathbf{12}} = 2N/N_0 = 7.55 \times 10^{-3}$ (3)

For the max of the absorption of **12** is 422 nm, $N_0 = 2.12 \times 10^{17}$ s^{-1}, $N = 5.77 \times 10^{14}$ s^{-1}, $AQY_{\mathbf{12}} = 5.44 \times 10^{-3}$

2. Synthetic procedures

Synthesis of trimethyl(pyren-1-ylethynyl)silane 3.

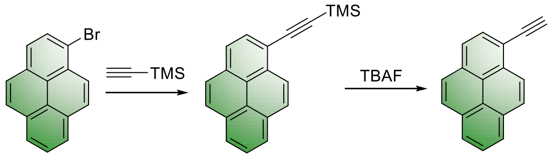

I   3

To a degassed solution of 1-bromopyrene (5.62 g, 20 mmol) in dry Et_3N (60 mL), were successively added $Pd(PPh_3)_2Cl_2$ (700 mg, 5 %), CuI (380 mg, 10 %) and ethynyltrimethylsilane (3.2 mL, 22 mmol). The reaction mixture was stirred at 70 °C for 20 h. The solvent was evaporated under reduced pressure and the crude material was purified through silica gel column chromatography (Eluent: cyclohexane) to give **I** as yellow solid (5.12 g, 86%). ^{1}H NMR (400 MHz, $CDCl_3$) δ: 8.54-8.52 (d, J = 8.0 Hz, 1H), 8.17-8.10 (m, 4H), 8.03-7.94 (m, 4H), 0.40 (s, 9H); ^{13}C NMR (100 MHz, $CDCl_3$) δ: 132.19, 131.29, 131.10, 130.93, 129.85, 128.33, 128.15, 127.14, 126.13, 125.59, 125.51, 125.45, 124.31, 124.25, 124.14, 117.50, 104.07, 100.14, 0.20. The ^{1}H and ^{13}C NMR data are consistent with the reported ones. [1]

To a solution of **I** (4.47 g, 15 mmol) in THF (50 mL), was added TBAF (1 mol/L in THF, 30 mL). The suspension was stirred at r.t. for 2 h. H_2O was added and the aqueous phase was extracted with CH_2Cl_2. The organic phase was washed with brine, dried and concentrated under reduced pressure to give **3** as brown crystals. (2.37 g, 70%.) ^{1}H NMR (400 MHz, $CDCl_3$) δ: 8.56-8.54 (d, J = 8.0 Hz, 1H), 8.19-8.12 (m, 4H), 8.06-7.97 (m, 4H), 3.61 (s, 1H); ^{13}C NMR (100 MHz, $CDCl_3$) δ: 132.48, 131.58, 131.15, 130.96, 130.15, 128.56, 128.41, 127.18, 126.29, 125.75, 125.69, 125.30, 124.39, 124.31, 124.17, 116.48, 82.79, 82.65. The ^{1}H and ^{13}C NMR data are consistent with the reported ones. [1]

Synthesis of 1-(*p*-tolylethynyl)pyrene 6.

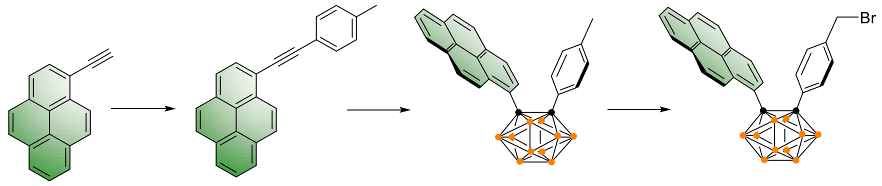

3   4   5   6

To a solution of **3** (2.26 g, 10 mmol) and 1-iodo-4-methylbenzene (2.4 g, 11 mmol) in CH_3CN (40 mL), was added Et_3N (40 mL). The solution was degassed under argon and CuI (114 mg, 6%) and $Pd(PPh_3)_4$ (345 mg, 3%) were successively added. The reaction mixture was stirred at 70 °C for 16 h under argon. H_2O was added and the aqueous phase was extracted with CH_2Cl_2. The organic phase was washed with brine, dried and concentrated under reduced pressure. Through silica gel column chromatography (Eluent: PE/EtOAc 10:1) to give **4** as orange solid. (2.56 g, 81%). M. p.: 99-101 °C. ^{1}H NMR (400 MHz, $CDCl_3$) δ: 8.63-8.60 (d, *J* = 12.0 Hz, 1H), 8.15-8.09 (m, 4H), 8.05-7.93 (m, 4H), 7.61-7.59 (d, *J* = 8.0 Hz, 2H), 7.21-7.19 (d, *J* = 8.0 Hz, 2H), 2.38 (s, 3H); ^{13}C NMR (100 MHz, $CDCl_3$) δ: 138.62, 131.84, 131.65, 131.28, 131.13, 131.12, 129.56, 129.32, 128.25, 128.05, 127.27, 126.21, 126.02, 125.62, 125.58, 125.52, 124.56, 124.52, 124.36, 120.54, 118.09, 95.42, 88.12, 21.66. HRMS calcd for $C_{25}H_{17}$ $[M+H]^+$ 317.1325; found: 317.1329.

Decaborane (0.71 g, 6.35 mmol) was dissolved in 50 mL toluene and *N*,*N*-dimethylaniline (0.23 mL, 1.82 mmol) under Ar. The mixture was stirred at room temperature for 2 h. **4** (1.58 g, 5 mmol) was added, and the mixture was additionally refluxed for 1 d. After cooling to room temperature, insoluble products were removed by filtration, and the solvent was evaporated. The residue was purified by column chromatography on a silica gel (PE/CH_2Cl_2 v/v = 10:1) to give a yellow powder **5** (0.98 g, 45%), M. p.: 246-248 °C. ^{1}H NMR (400 MHz, $CDCl_3$) δ: 9.35-9.33 (d, *J* = 8.0 Hz, 1H), 8.41-8.38 (d, *J* = 12.0 Hz, 1H), 8.24-8.17 (m, 3H), 8.06-8.01 (m, 2H), 7.88-7.84 (m, 2H), 7.21-7.19 (d, *J* = 8.0 Hz, 2H), 6.66-6.64 (d, *J* = 8.0 Hz, 2H), 2.82 (s, 3H); ^{13}C NMR (100 MHz, $CDCl_3$) δ: 140.26, 132.87, 131.69, 131.15, 131.09, 130.11, 129.80, 129.42, 128.86, 128.49, 128.16, 126.94, 126.45, 125.94, 125.42, 124.32, 124.20, 123.99, 122.68, 89.87, 88.99, 20.80. HRMS calcd for $C_{25}H_{27}B_{10}$ $[M+H]^+$ 437.3038; found: 437.3044.

To a solution of **5** (0.79 g, 1.8 mmol.) in carbon tetrachloride (40 ml), added azobisisobutyro nitrile (AIBN) (23.6 mg, 8 %.) as a radical generator. To this suspension added *N*-bromosuccinimide (NBS) (336 mg, 1.1 equiv.) and refluxed at 100 ^{o}C for 12 h. The reaction mixture was filtered and concentrated to get a dull yellow solid compound, which was washed with hexane (50 mL) and CH_3CN (10 mL) and Et_2O (5 mL), dried under high vacuum to obtain **6**/**5** = 3/2. It's noted, the compound **6** decomposes

easily in columns or solvent with water, so we used the mixture **6** and **5** directly for the next step. HRMS calcd for $C_{25}H_{26}B_{10}Br$ $[M+H]^+$ 515.2143; found: 515.2130.

Synthesis of 1-((4-(bromomethyl)phenyl)ethynyl)pyrene 10.

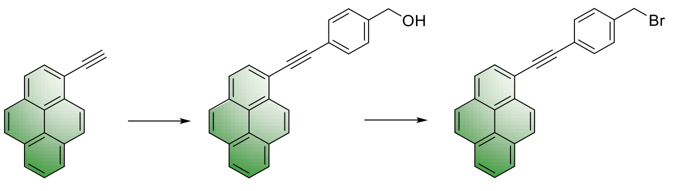

To a solution of **3** (2.26 g, 10 mmol) and (4-iodophenyl)methanol (2.34 g, 11 mmol) in CH_3CN (40 mL), was added Et_3N (40 mL). The solution was degassed under argon and CuI (114 mg, 6%) and $Pd(PPh_3)_4$ (345 mg, 3%) were successively added. The reaction mixture was stirred at 70 °C for 16 h under argon. H_2O was added and the aqueous phase was extracted with CH_2Cl_2. The organic phase was washed with brine, dried and concentrated under reduced pressure. Through silica gel column chromatography (Eluent: PE/EtOAc 4:1) to give **9** as orange solid. (2.85 g, 86%). M. p.: 158-160 °C. ^{1}H NMR (400 MHz, $CDCl_3$) δ: 8.67-8.65 (d, *J* = 8.0 Hz, 1H), 8.23-8.17 (m, 4H), 8.13-8.07 (m, 4H), 7.73-7.71 (d, *J* = 8.0 Hz, 2H), 7.44-7.42 (d, *J* = 8.0 Hz, 2H), 4.77-4.76 (d, *J* = 4.0 Hz, 2H); ^{13}C NMR (100 MHz, $CDCl_3$) δ: 141.14, 137.60, 135.23, 131.89, 131.27, 131.08, 129.63, 128.37, 128.19, 127.28, 126.98, 126.27, 125.66, 125.61, 125.55, 124.57, 124.51, 124.34, 122.78, 117.74, 94.96, 88.75, 65.06. HRMS calcd for $C_{25}H_{17}O$ $[M+H]^+$ 333.1274; found: 333.1264.

A solution of the **9** (1.66 g, 5 mmol) in dioxane (40 mL) was added PBr_3 (0.52 mL, 5.5 mmol) and the mixture was stirred for 2 h at room temperature. The solvent was removed under reduced pressure and the product was extracted with DCM, washed with water. The organic phase was dried over $MgSO_4$ and the solvent was evaporated. The residue was purified by flash column chromatography with PE/DCM (v/v =10/1) as an eluent to afford **10** as yellow solid (1.04 mg, 53 %). M. p.: 176-178 °C. ^{1}H NMR (400 MHz, $CDCl_3$) δ: 8.66-8.63 (d, *J* = 12.0 Hz, 1H), 8.25-8.18 (m, 4H), 8.15-8.02 (m, 4H), 7.70-7.68 (d, *J* = 8.0 Hz, 2H), 7.47-7.45 (d, *J* = 8.0 Hz, 2H), 4.55 (s, 2H); ^{13}C NMR (100 MHz, $CDCl_3$) δ: 137.91, 132.07, 131.96, 131.39, 131.25, 131.06, 129.66, 129.25, 128.45, 128.29, 127.27, 126.31, 125.73,

125.68, 125.47, 124.57, 124.49, 124.31, 123.71, 117.51, 94.60, 89.60, 33.16. HRMS calcd for $C_{25}H_{16}Br$ $[M+H]^+$ 395.0430; found:395.0398.

Synthesis of 7 and 8.

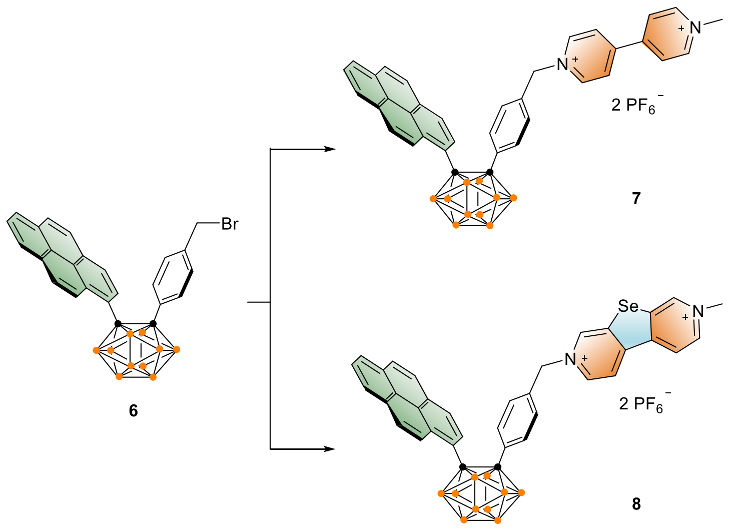

6 (257 mg, 0.5 mmol) and 1-methyl-[4,4'-bipyridin]-1-ium iodide (119.2 mg, 0.4 mmol) were dissolved in dried and degassed DMF (10 mL) and heated to 70 °C for 2 d while stirring. The solvent was removed via vacuum and washed with dichloromethane (3 × 30 mL) for at least 3 times. The precipitate was added to saturated ammonium hexafluorophosphate solution and the suspension was stirred overnight at room temperature. The precipitate was collected and dried under high vacuum to obtain **7** (165 mg, 46%). M. p.: 257-259 °C. ^{1}H NMR (400 MHz, DMSO-d^6) δ: 9.24-9.20 (m, 5H), 8.62-8.61 (m, 5H), 8.48-8.37 (m, 3H), 8.27-8.25 (d, *J* = 8.0 Hz, 1H), 8.8.19-8.10 (m, 3H), 7.57-7.55 (d, *J* = 8.0 Hz, 2H), 7.19-7.17 (d, *J* = 8.0 Hz, 2H), 5.62 (s, 2H), 4.42 (s, 3H); ^{13}C NMR (150 MHz, DMSO-d^6) δ: 148.48, 147.41, 145.96, 145.17, 135.98, 132.15, 131.63, 130.44, 130.30, 130.02, 129.51, 129.30, 128.60, 128.51, 127.96, 126.47, 126.31, 126.23, 125.77, 125.43, 123.99, 123.79, 122.64, 122.27, 120.61, 88.78, 88.33, 64.29, 47.44. HRMS calcd for $C_{36}H_{36}B_{10}N_2$ $[M]^{2+}$ 606.3809; found: 606.3839.

6 (257 mg, 0.5 mmol) and 2-methylselenopheno[2,3-c:5,4-c']dipyridin-2-ium iodide (150.4 mg, 0.4 mmol) were dissolved in dried and degassed DMF (10 mL) and heated to 70 °C for 2 d while stirring. The solvent was removed via vacuum and washed with dichloromethane (3 × 30 mL) for at least 3 times. The precipitate was added to saturated ammonium hexafluorophosphate solution and the

suspension was stirred overnight at room temperature. The precipitate was collected and dried under high vacuum to obtain **8** (196 mg, 51%). M. p.: 251-253 °C. ^{1}H NMR (400 MHz, DMSO-d^6) δ: 9.97-9.92 (d, *J* = 20 Hz, 2H), 9.22-9.19 (d, *J* = 12 Hz, 5H), 8.62-8.61 (d, *J* = 8 Hz, 1H), 8.42-8.24 (m, 3H), 8.08-7.95 (m, 4H), 7.60-7.59 (d, *J* = 4.0 Hz, 2H), 7.27-7.25 (d, *J* = 8.0 Hz, 2H), 5.78 (s, 2H), 4.54 (s, 3H); ^{13}C NMR (150 MHz, DMSO-d^6) δ: 146.84, 146.29, 145.95, 145.37, 144.19, 143.59, 141.32, 140.51, 136.95, 133.12, 132.71, 131.63, 131.51, 130.93, 130.51, 130.21, 129.57, 129.44, 127.40, 127.11, 126.70, 125.06, 124.76, 124.56, 123.89, 123.57, 123.27, 121.61, 89.96, 89.50, 65.39, 49.54. HRMS calcd for $C_{36}H_{34}B_{10}N_2Se$ $[M]^{2+}$ 684.2818; found: 684.2810.

Synthesis of 11 and 12.

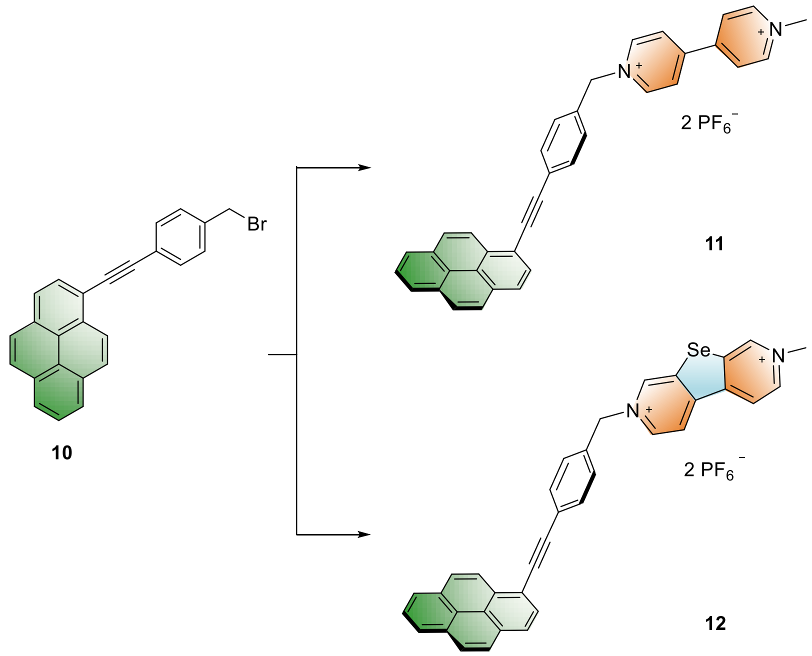

10 (197 mg, 0.5 mmol) and 1-methyl-[4,4'-bipyridin]-1-ium iodide (119.2 mg, 0.4 mmol) were dissolved in dried and degassed DMF (10 mL) and heated to 70 °C for 2 d while stirring. The solvent was removed via vacuum and washed with dichloromethane (3 × 30 mL) for at least 3 times. The precipitate was added to saturated ammonium hexafluorophosphate solution and the suspension was stirred overnight at room temperature. The precipitate was collected and dried under high vacuum to obtain **11** (214 mg, 69%). M. p.: 235-237 °C. ^{1}H NMR (400 MHz, DMSO-d^6) δ 9.57-9.56 (d, *J* = 4.0 Hz, 2H), 9.29-9.28 (d, *J* = 4.0 Hz, 2H), 8.83-8.74 (m, 4H), 8.64-8.62 (d, *J* = 8.0 Hz, 1H), 8.43-8.14 (m, 8H),

7.93-7.91 (d, J = 8.0 Hz, 2H), 7.77-7.75 (d, J = 8.0 Hz, 2H), 6.04 (s, 2H), 4.45 (s, 3H); ^{13}C NMR (100 MHz, DMSO-d^6) δ: 149.74, 148.67, 147.07, 146.35, 135.05, 132.77, 131.67, 131.23, 130.93, 130.20, 129.93, 129.49, 129.09, 127.72, 127.59, 127.35, 126.67, 126.59, 125.47, 125.20, 124.10, 123.82, 116.71, 94.85, 89.95, 63.57, 48.55. HRMS calcd for $C_{36}H_{26}N_2$ $[M]^{2+}$ 486.2085; found: 486.2076.

10 (197 mg, 0.5 mmol) and 2-methylselenopheno[2,3-c:5,4-c']dipyridin-2-ium iodide (150.4 mg, 0.4 mmol) were dissolved in dried and degassed DMF (10 mL) and heated to 70 °C for 2 d while stirring. The solvent was removed via vacuum and washed with dichloromethane (3 × 30 mL) for at least 3 times. The precipitate was added to saturated ammonium hexafluorophosphate solution and the suspension was stirred overnight at room temperature. The precipitate was collected and dried under high vacuum to obtain **12** (290 mg, 85%). M. p.: 240-242 °C. ^{1}H NMR (400 MHz, DMSO-d^6) δ: 9.57-9.56 (d, J = 4.0 Hz, 2H), 9.29-9.28 (d, J = 4.0 Hz, 2H), 8.83-8.74 (m, 4H), 8.64-8.62 (d, J = 8.0 Hz, 1H), 8.43-8.14 (m, 8H), 7.93-7.91 (d, J = 8.0 Hz, 2H), 7.77-7.75 (d, J = 8.0 Hz, 2H), 6.04 (s, 2H), 4.45 (s, 3H); ^{13}C NMR (100 MHz, DMSO-d^6) δ: 149.74, 148.67, 147.07, 146.35, 135.05, 132.77, 131.67, 131.23, 130.93, 130.20, 129.93, 129.49, 129.09, 127.72, 127.59, 127.35, 126.67, 126.59, 125.47, 125.20, 124.10, 123.82, 116.71, 94.85, 89.95, 63.57, 48.55. HRMS calcd for $C_{36}H_{24}N_2Se_2$ $[M]^{2+}$ 564.1094; found: 564.1021.

3. UV−Vis spectra

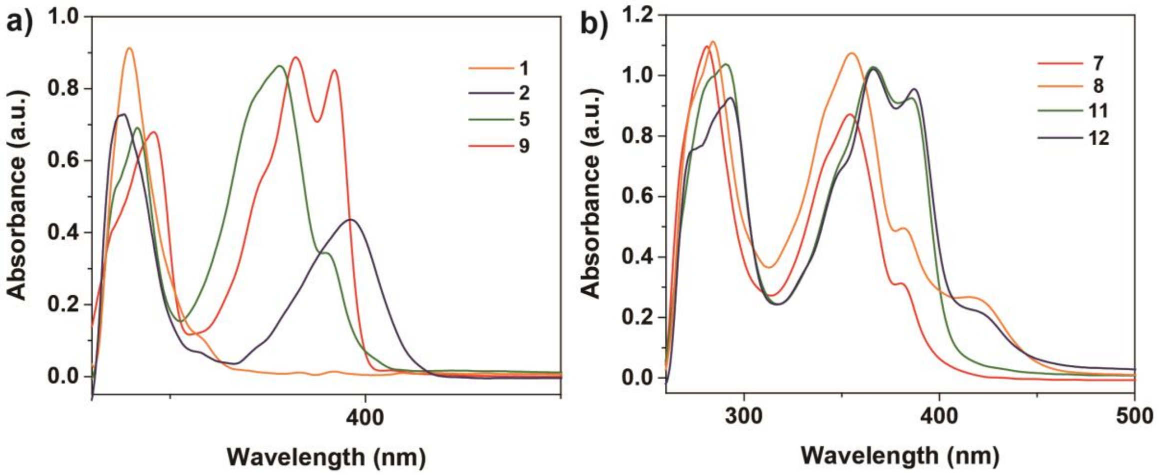

Figure S1. UV/Vis spectra of (a) **1**, **2**, **5**, **9** amd (b) **7, 8, 11, 12** in DMF.

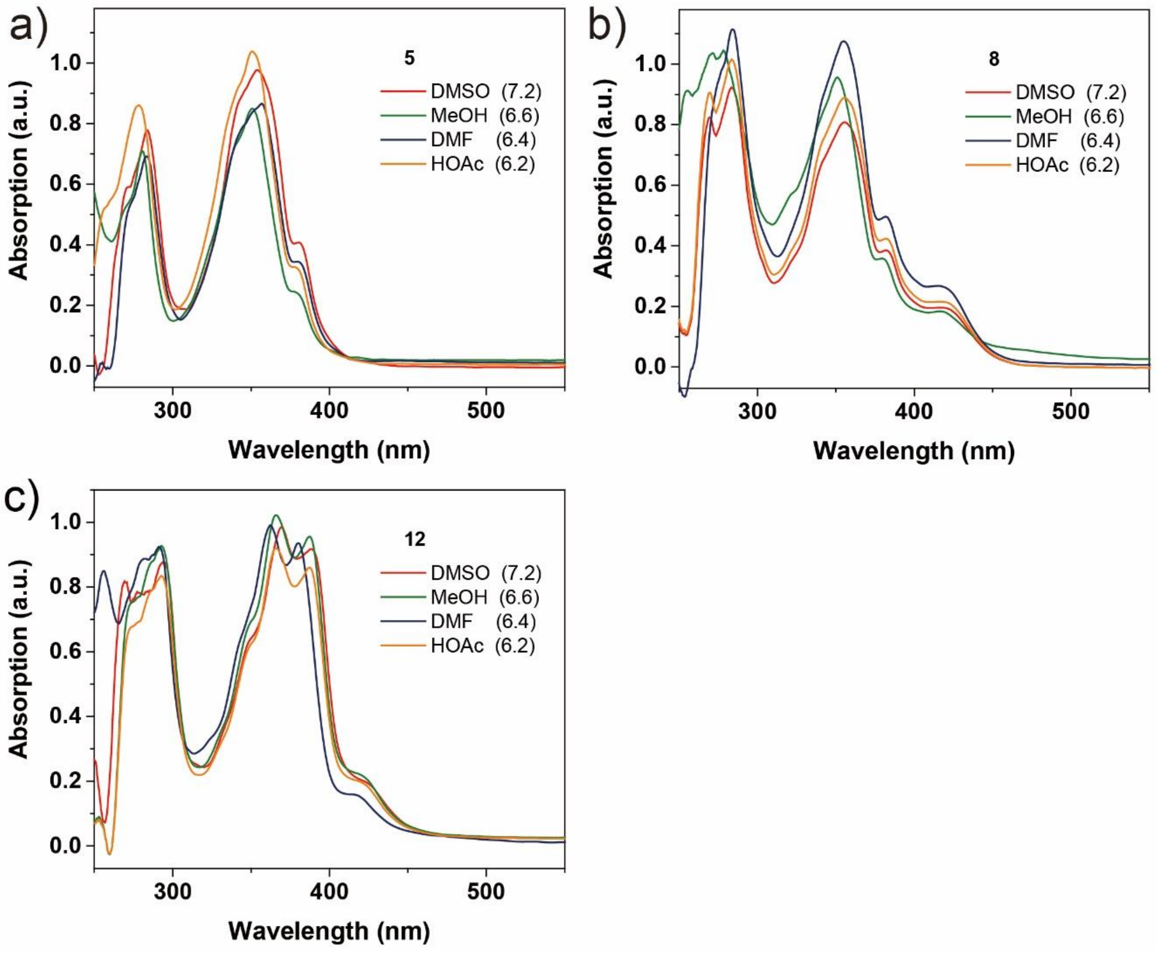

Figure S2. UV/Vis spectra of (a) **5**, (b) **8** and (c) **12** in different solutions.

4. The cyclic voltammogram

Cyclic Voltammetry was performed under Ar atmosphere with a CHI 600E potentiostation in a solution of anhydrous DMF with 0.1 M tetrabutylammonium hexafluorophosphate as supporting electrolyte, at a scan rate of 10, 20, 50, 100, 200, 500 mV s^{-1}. A glassy carbon was used as working electrode; a platinum wire was used as the auxiliary electrode, and an Ag/AgCl was used as reference electrode. Under these conditions, $E_{1/2}$ = 0.56 V for the $FeCp_2^+$/ $FeCp_2$ couple.

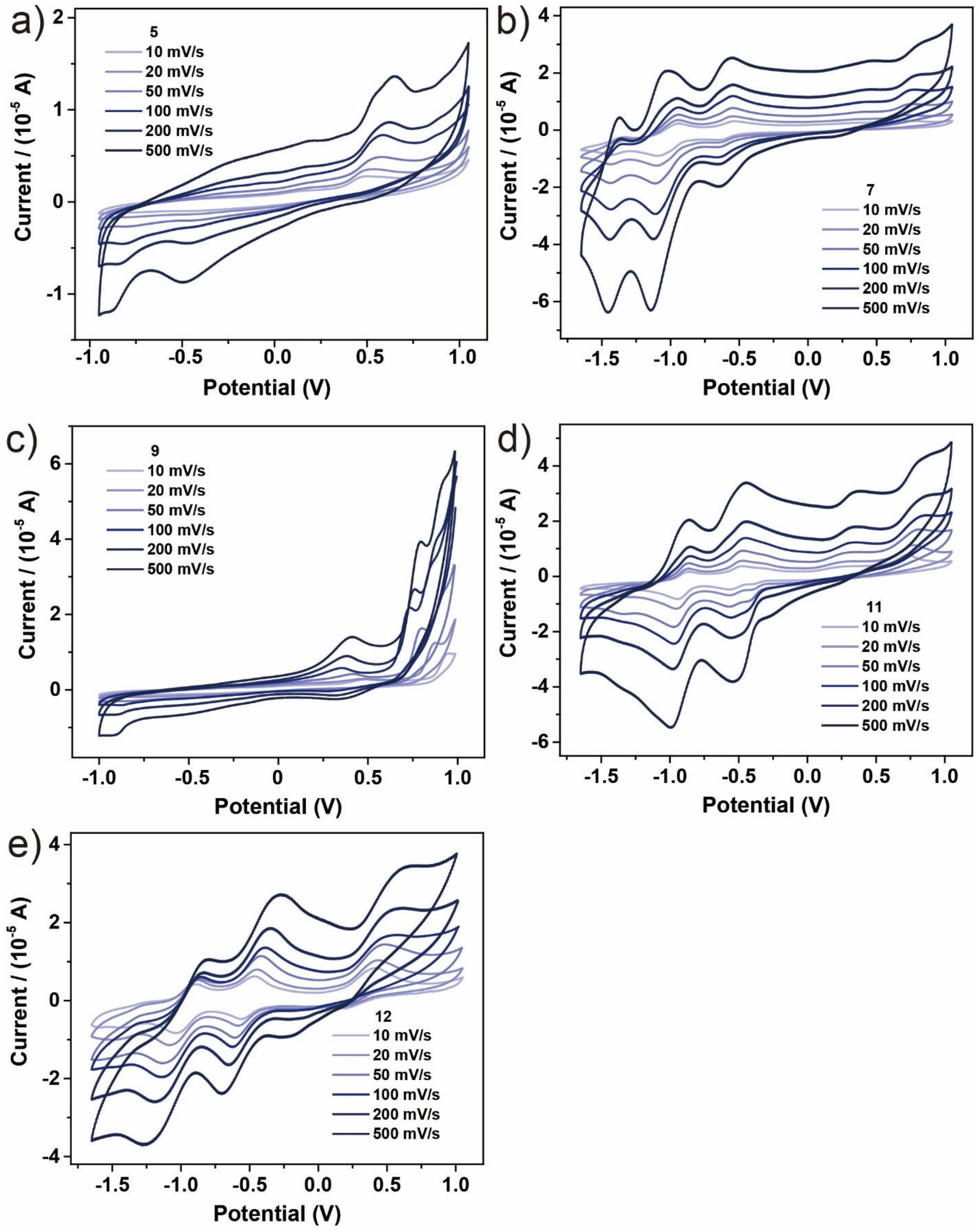

Figure S3. Cyclic voltammograms of **5**, **7-9**, **11,12** recorded in DMF (10^{-3} M)

Table S1. Optical and Electronic Properties of **7, 8, 11, 12**.

Compound	λ_{max} [nm]	Electrochemical		LUMO (eV)[a]		HOMO (eV)		Egap (eV)[b]	
		E_{red} (V)	E_{ox} (V)	(Exp)[a]	(Calc)[b]	(Exp)	(Calc)[b]	(Exp)[c]	(Calc)[b]
**7**	382, 354, 280	-0.46, -0.83, -1.32	0.14, 0.45	-4.34	-3.73	-6.95	-6.24	2.61	2.51
**8**	419, 381, 355, 283	-0.58, -1.04, -1.42	0.14, 0.46	-4.22	-3.82	-6.52	-6.01	2.30	2.19
**11**	386, 366, 291	-0.64, -1.14	0.15	-4.16	-3.74	-6.69	-5.94	2.53	2.20
**12**	421, 387, 366, 292	-0.62, -1.16	0.21	-4.18	-3.80	-6.51	-5.93	2.33	2.13

[a] Energy levels vs vacuum level were calculated from CV data and from the optically determined energy gap. [b] Theoretical calculations have been carried out by using the GAUSSIAN09 suite of programs. [c] Energy gap values were calculated from the absorption spectra.

5. Photoluminescence (PL) spectra

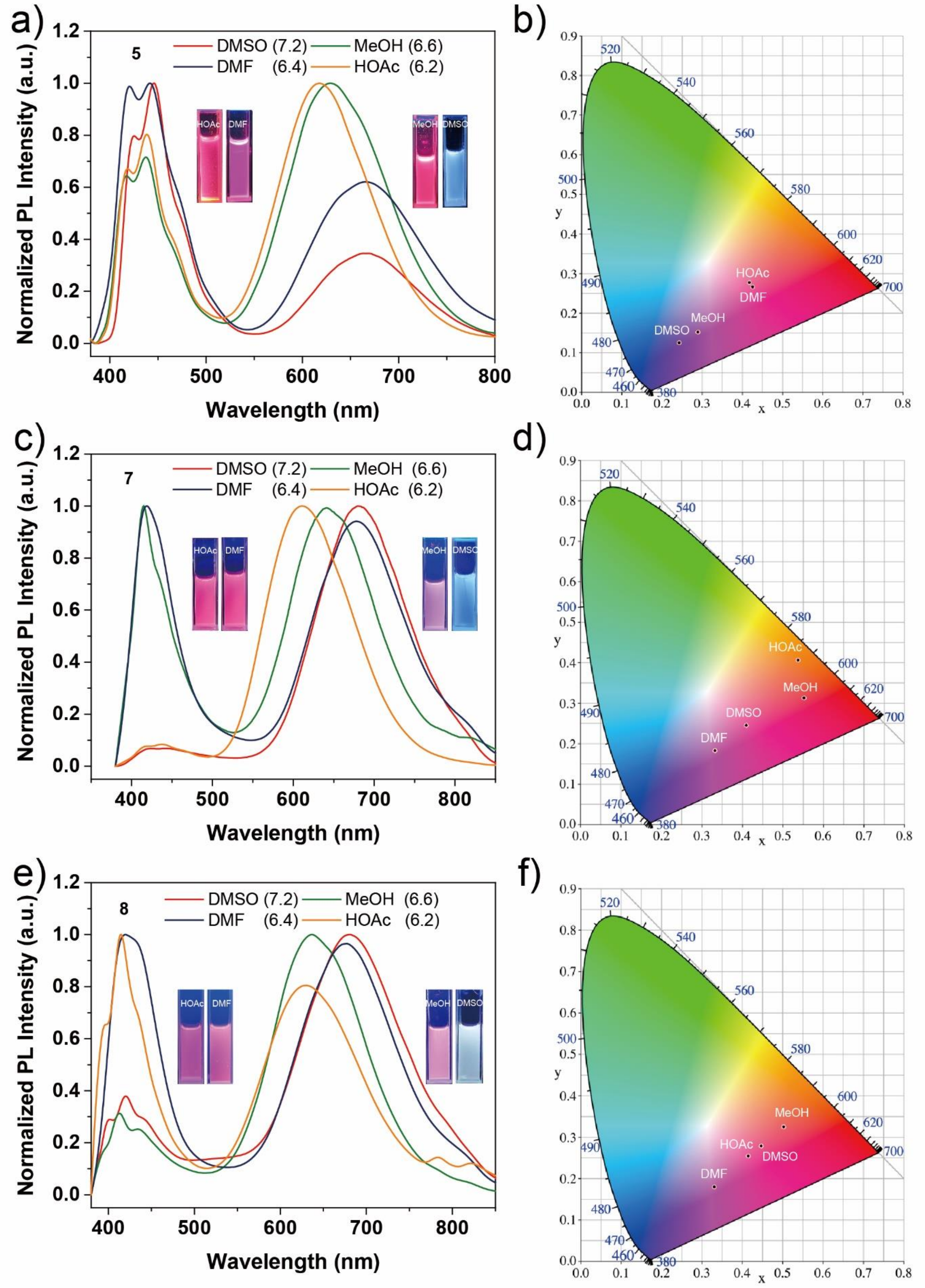

Figure S4. Photoluminescence (PL) spectra of (a) for **5,** (c) for **7** and (e) for **8** ($5x10^{-5}$ M) in different solvent. CIE chromaticity of (b) for **5,** (d) for **7** and (f) for **8.**

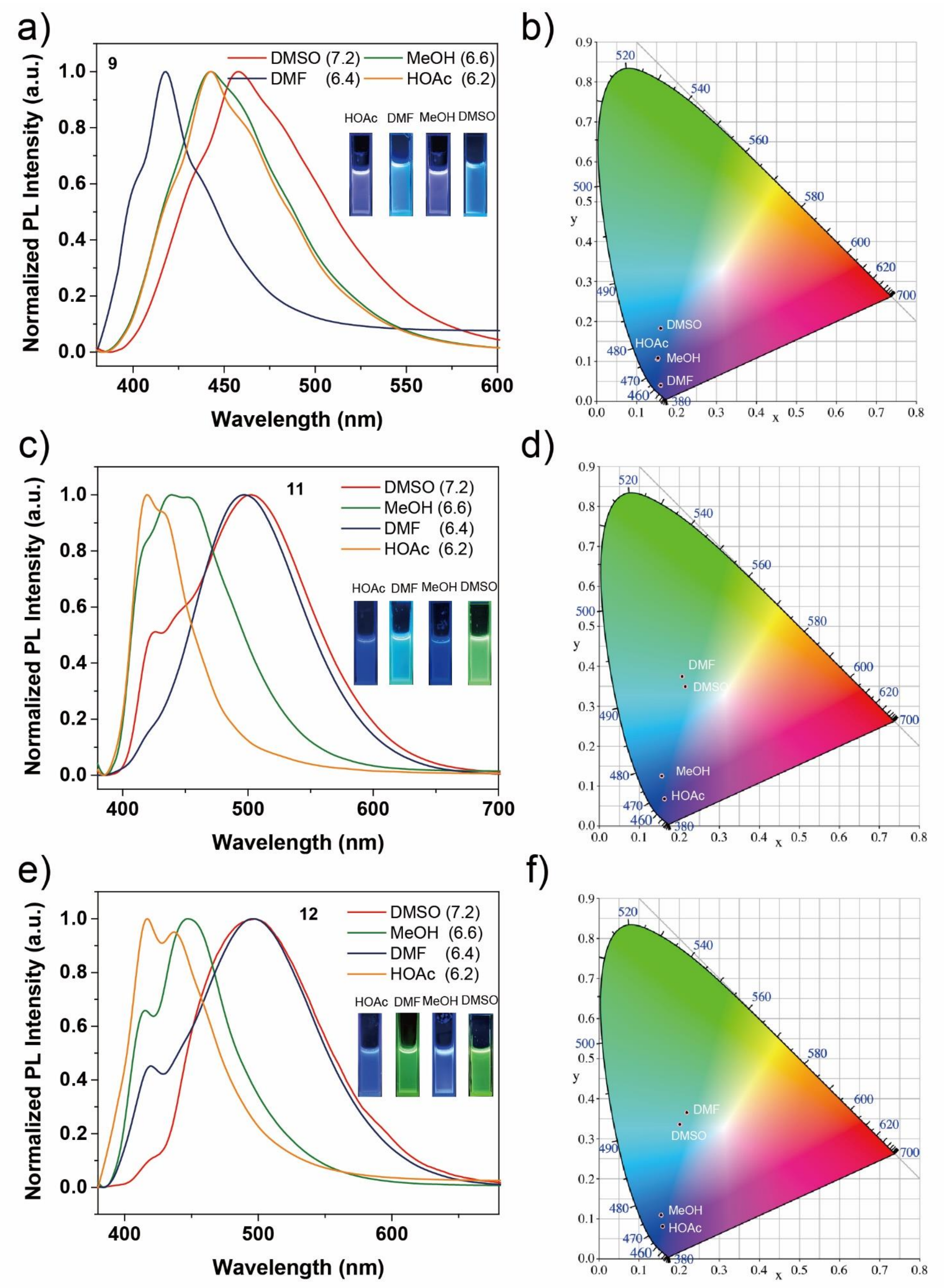

Figure S5. Photoluminescence (PL) spectra of (a) for **9,** (c) for **11** and (e) for **12** ($5x10^{-5}$ M) in different solvent. CIE chromaticity of (b) for **9,** (d) for **11** and (f) for **12.**

Table S2. Summarized emission data, photoluminescence lifetimes (τ), quantum yield and electron-transfer constant k_{ET} of **5**, **7, 8, 9, 11, 12** in solution (DMF) and solid state.

Compound		Wavelength (nm)	τ_1 (ns)	τ_2 (ns)	QY	k_{ET} (s^{-1}) [a]
**5**	solution	707	1.61	0.49	4.20%	-
	solid	591	5.32	1.98	55.81%	-
**7**	solution	711	1.35	4.92	0.26%	7.22×10^{9}
	solid	650	0.96	3.48	0.75%	1.01×10^{10}
**8**	solution	720	1.36	3.26	0.16%	1.20×10^{10}
	solid	676	1.32	2.45	0.42%	1.81×10^{10}
**9**	solution	435	1.27	0.62	96.42%	-
	solid	505	1.54	3.70	14.73%	-
**11**	solution	506	2.82	0.38	18.08%	2.29×10^{9}
	solid	600	3.67	6.89	0	-
**12**	solution	503	2.66	0.41	13.18%	3.34×10^{9}
	solid	576	0.76	6.67	0	-

[a] electron-transfer process k_{ET} was calculated with the following equation $k_{\mathrm{ET}} = \frac{\left(\frac{\phi_{\mathrm{ref}}}{\phi}\right)-1}{\tau_{\mathrm{ref}}}$ here Φ_{ref} and Φ are the relative fluorescence quantum yields of the model compound **5** or **9,** respectively, and τ_{ref} is the fluorescence lifetime of the model compound **5** or **9.**[4]

6. Electrochromism and electrofluorochromic

In the solution-based ECD, Fluorine-doped tin oxide (FTO)-coated glass (~ 10 Ω/sq) was utilized as the electrodes and **7, 8, 11, 12** were used as active component. The two pieces of FTO glass were sealed together with a UV-cured gasket with 50 μm-thick intervals introduced by Baumgartner group.[5]

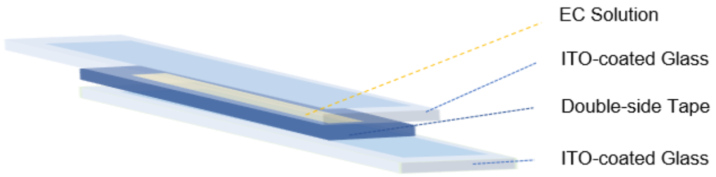

Figure S6. Device fabrication of solution-based ECDs.

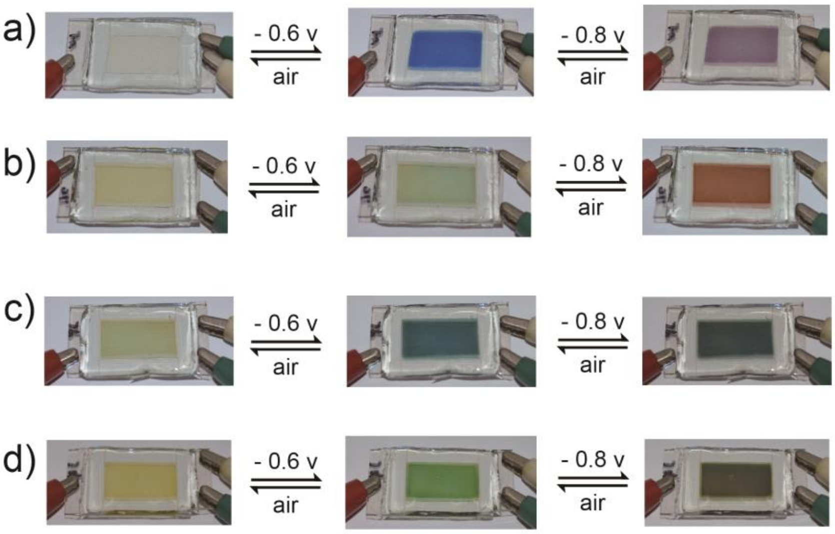

Figure S7. (a-d) Solution-based electrochromic device with **7, 8, 11, 12** (no electrolyte).

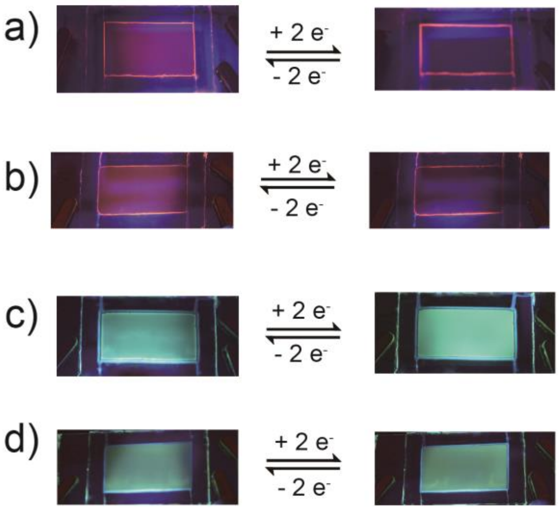

Figure S8. (a-d) Solution-based electrofluorochromic device with **7, 8, 11, 12** under 365 nm light (no electroly

7. The spectroelectrochemistry

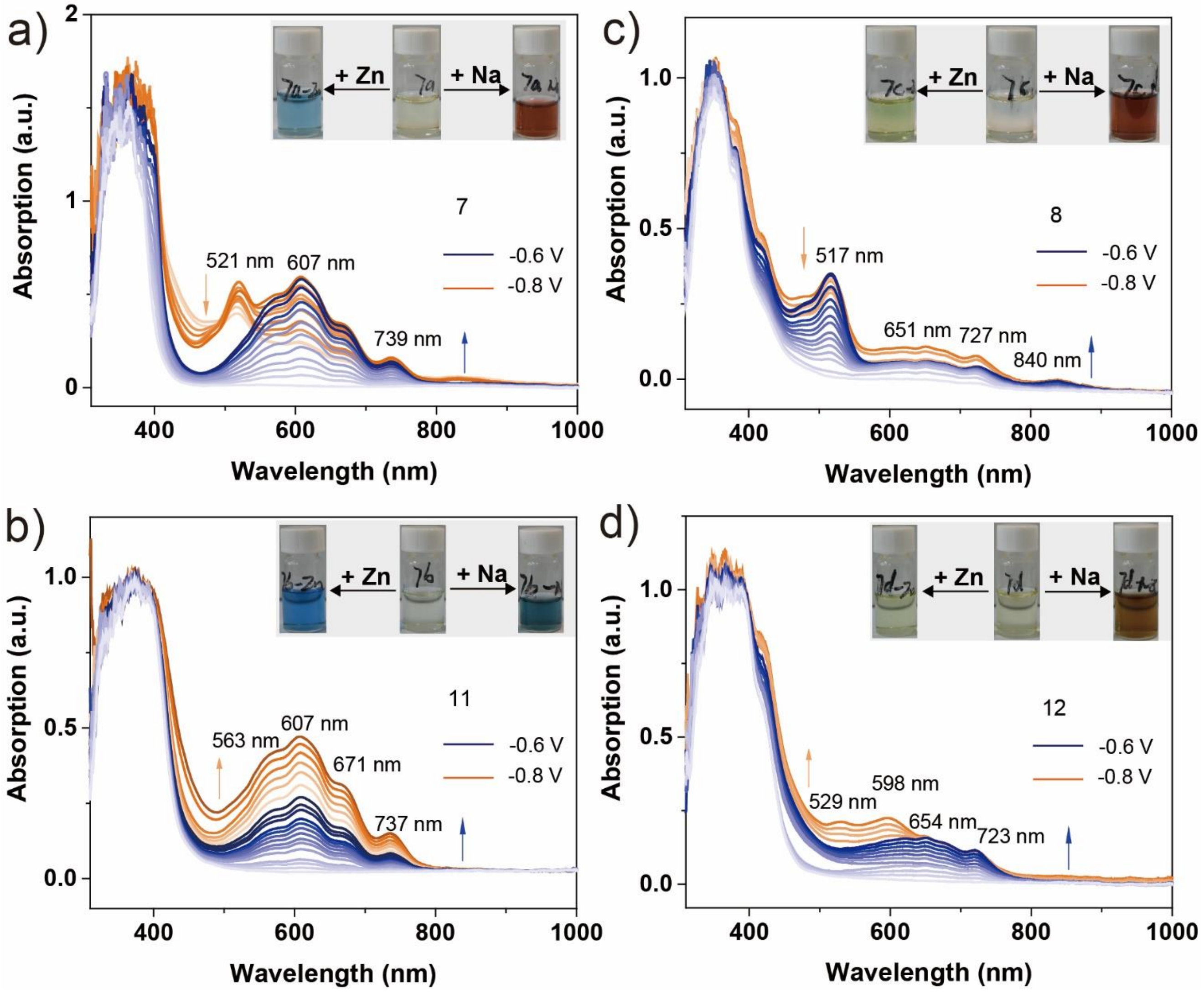

Figure S9. (a-d) UV-Vis Spectroelectrochemistry of **7, 8, 11, 12** for first reduction and second reduction.

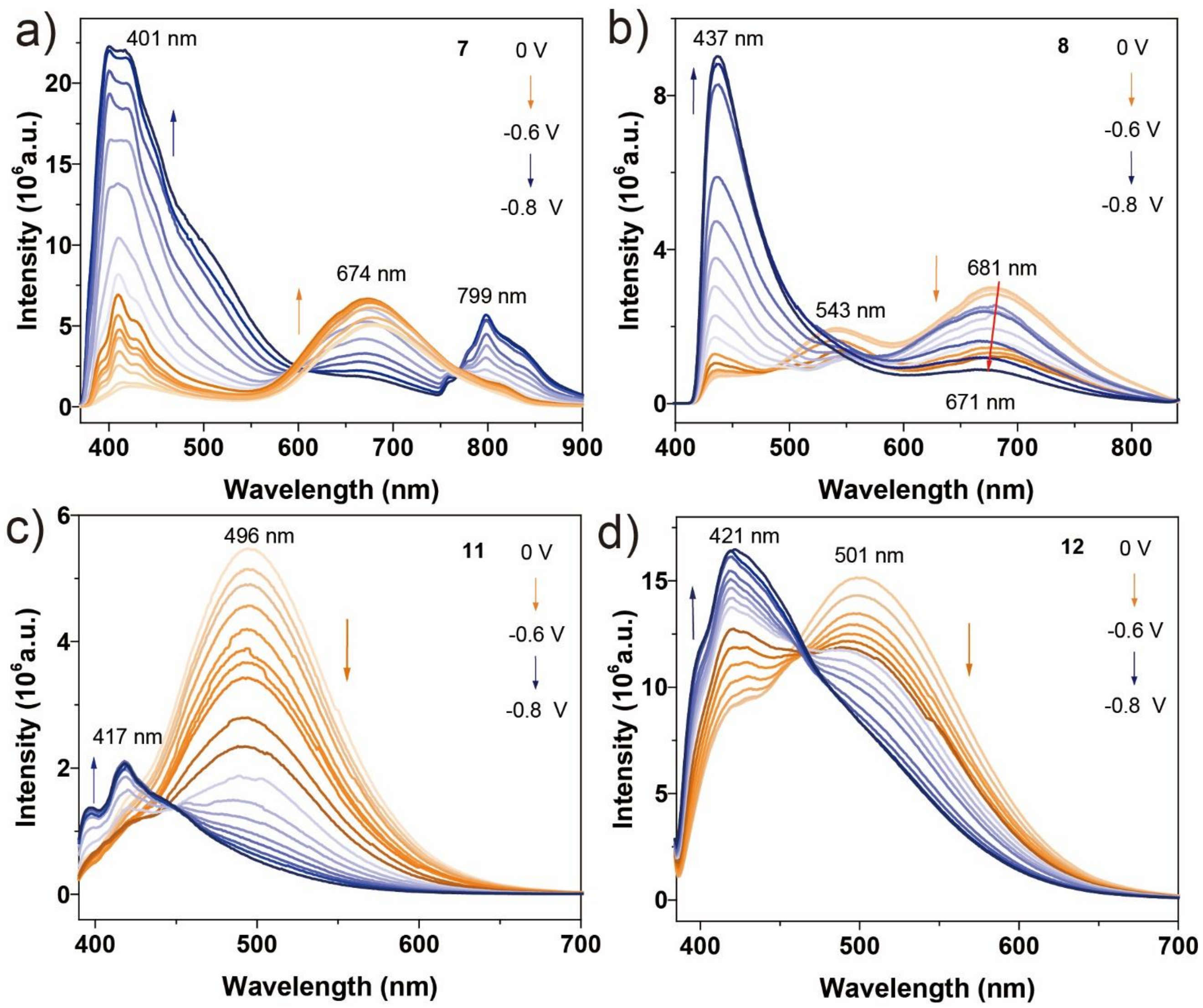

Figure S10. (a-d) PL Spectroelectrochemistry of **7, 8, 11, 12** for first reduction and second reduction and their solution-based

electrochromic.

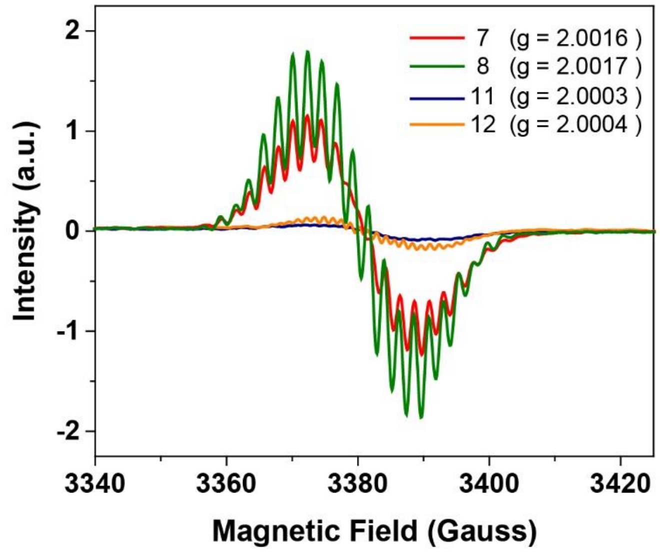

Figure S11. EPR spectrum of **7, 8, 11, 12** for Zn reduction state

8. TEM

The componds **7**, **8**, **11**, **12** were loaded into the water, and using ultrasound to disperse it; Then the solution was dripped onto the copper mesh; After the sample was air dried, the TEM was performed on an HT7700 operated at an acceleration voltage of 100 kV.

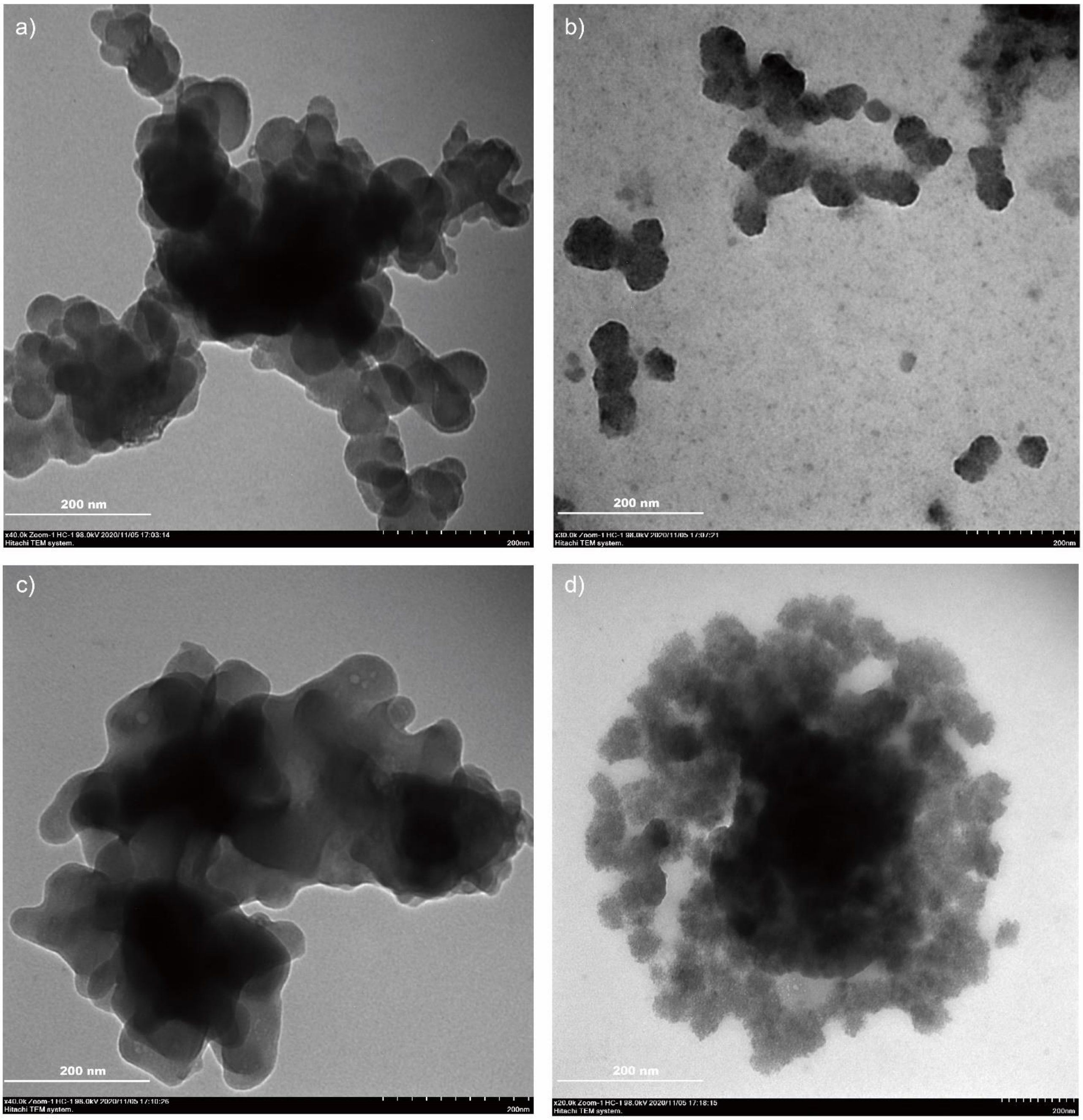

Figure S12. (a-d) TEM image of **7, 8, 11, 12** nanoparticles.

9. H_2 normalized curve

The system in which H_2 was 1.0 percent (volume fraction) and protected by Argon was chosen as the"standard H_2". 40 μL, 60 μL, 80 μL, 120 μL, 160 μL, 200 μL gas from the "standard H_2" was injected to GC respectively, every volume for three times. The curve figure was the H_2 peak area verse the number of moles of hydrogen, and the equation was fitted by origin.

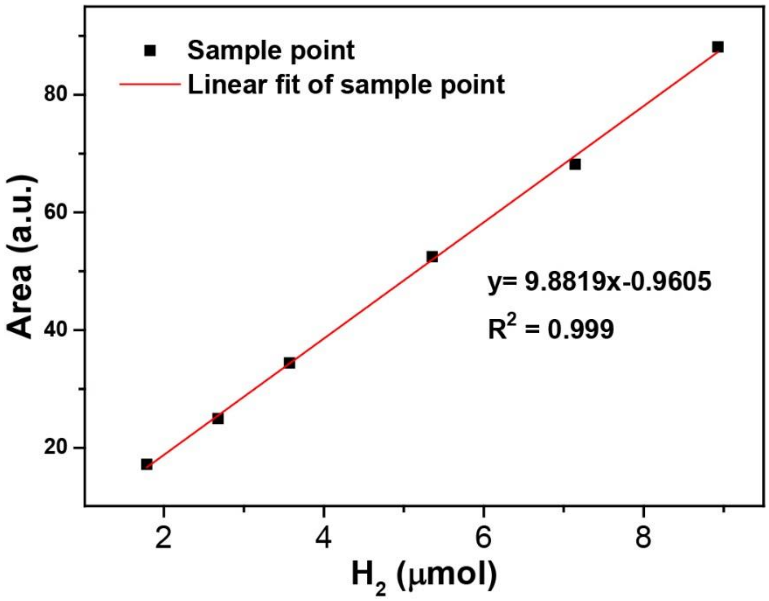

Figure S13. H_2 peak area verse the number of moles of hydrogen and polynomial fit of sample point to be H_2 normalized curve

10. Hydrogen generation under xenon lamp

The mixed aqueous solution containing **7** (2.5 mg), EDTA (100 mg) and PVP-Pt (2.5 mg) was sealed in the 20 mL Pyrex bottle with a rubber stopper. The solution was bubbled with Argon for 30 min, after that it was irradiated under Xenon lamp (λ > 400 nm) with light power of 100 mW. Then 200 μL upper gas of the reactor was injected to gas chromatography per hour to measure hydrogen generation. The production of the hydrogen was calculated according to the H_2 normalized curve (see our previous work).

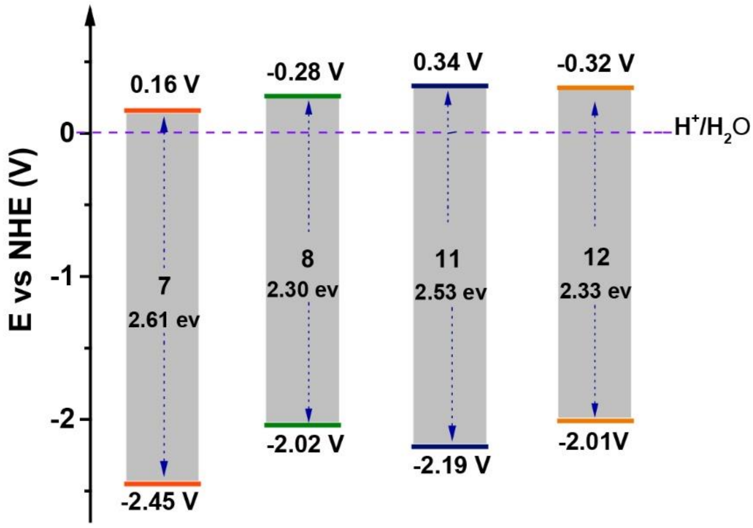

Figure S14. Experimental energy levels of the LUMO and HOMO orbitals for **7, 8, 11, 12**.

Table S3. Hydrogen generation activities of molecules **4**, **5**, and **9** are physically mixed with selenoviologen or viologen.

System	Total hydrogen generation[a] (μmol)	Hydrogen generation rate (mmol•h^{-1}•g^{-1})
selenoviologen	29.69 μmol	0.50
viologen	0	-
**4** + selenoviologen	28.99 μmol	0.50
**5** + selenoviologen	30.90 μmol	0.51
**9** + selenoviologen	29.39 μmol	0.50
**4** + viologen	trace	-
**5** + viologen	trace	-
**9** + viologen	trace	-

[a] The amount of hydrogen generation in 24 hours.

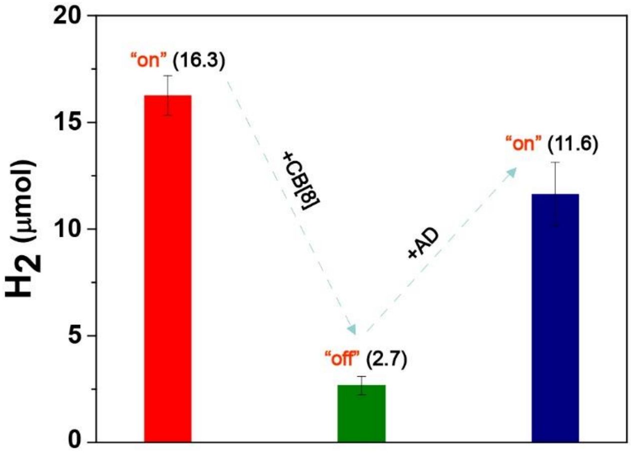

Figure S15. Controlled hydrogen generation for **8** using CB[8] and AD by supramolecular assembly and disassembly process.

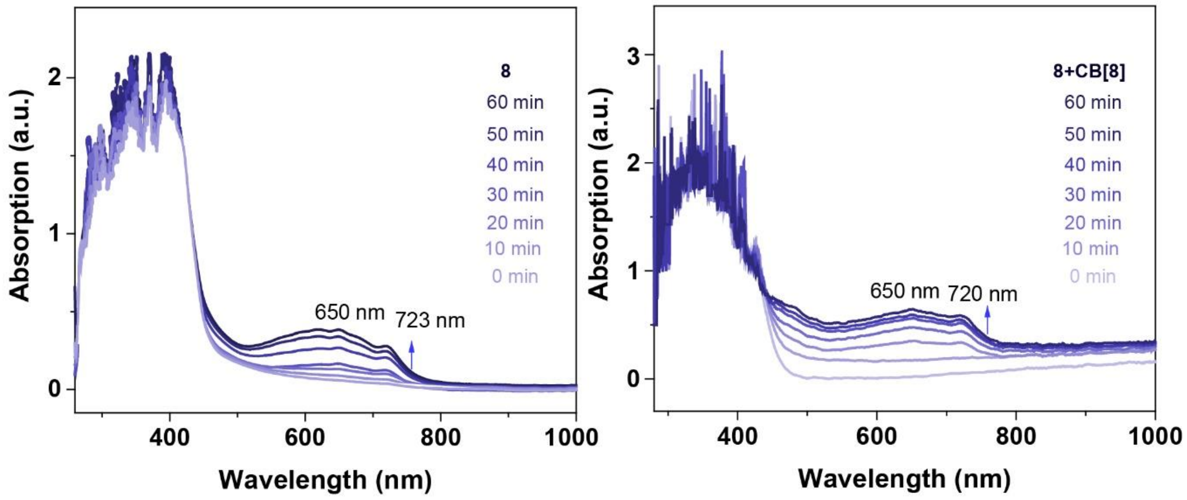

Figure S16. UV-Vis spectra of **8** and **8**+CB[8] under visible-light irradiation.

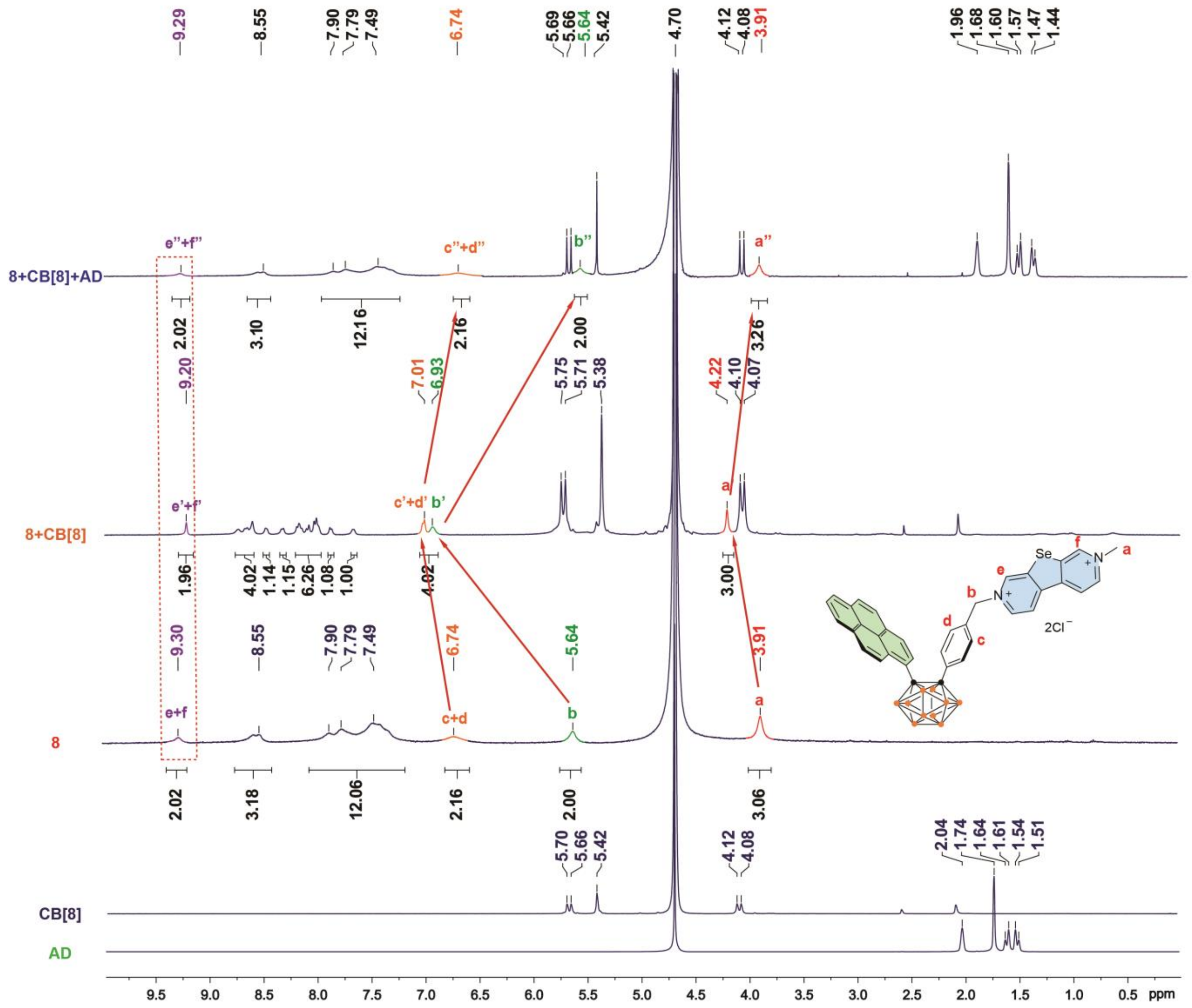

Figure S17. ^{1}H NMR spectra of **AD**, CB[8], **8**, **8** + CB[8] and **8** + CB[8] + AD in NaCl/D_2O (100mM) solution.

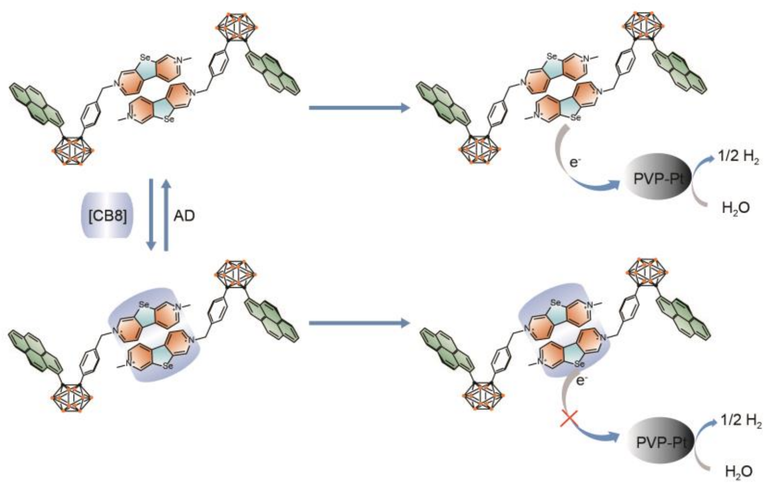

Figure S18. Supramolecular chemistry regulates hydrogen production

11. Reduction of phenylacetylene under xenon lamp

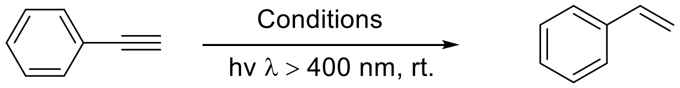

1 2

Table S4. Optimization of reaction conditions [a]

entry	Additive (mg)	EDTA(mg)	PVP-Pt (mg)	solvent	Yield (%) [f]
1	8 (2)	100	2	DMF/BS. (4/1)	-
2	8 (2)	100	2	CH_3CN/BS. (4/1)	-
3	8 (2)	100	2	DCM/BS. (4/1)	-
4	8 (2)	100	2	Cyclohexane/BS. (4/1)	5
**5**	8 (2)	100	2	MeOH/BS. (4/1)	-
**6**	8 (2)	100	2	Cyclohexane/BS. (3/1)	6
7	8 (2)	100	2	Cyclohexane/BS. (2/1)	18
8	8 (2)	100	2	Cyclohexane/BS. (1/1)	24
9	8 (2)	100	2	Cyclohexane/BS. (1/2)	10
**10**	**8 (5)**	**250**	**5**	**Cyclohexane/BS. (1/1)**	**37**
11	8 (5)	250	5	Cyclohexane/water	-
12 [b]	8 (5)	250	5	Cyclohexane/BS. (1/1)	-
13 [c]	8 (5)	250	5	Cyclohexane/BS. (1/1)	trace
14 [d]	8 (5)	250	5	Cyclohexane/BS. (1/1)	trace
15 [e]	8 (5)	250	5	Cyclohexane/BS. (1/1)	30

[a] Reaction conditions: **1** (0.21 mmol), organic solvent (2 mL), buffer solution (BS. pH = 5.0), hv λ > 400 nm, r.t., 24 h, [b] BS. pH = 3.6, [C] BS. pH = 4.5, [d] BS. pH = 6.0, [e] Aliquant336 (5 mg), [f] Yields were determined by ^{1}H NMR analysis of the crude reaction mixture with 1,3,5-trimethoxybenzene as an internal standard. Entry in bold highlights optimized reaction conditions.

12. The mechanism of photocatalytic reaction

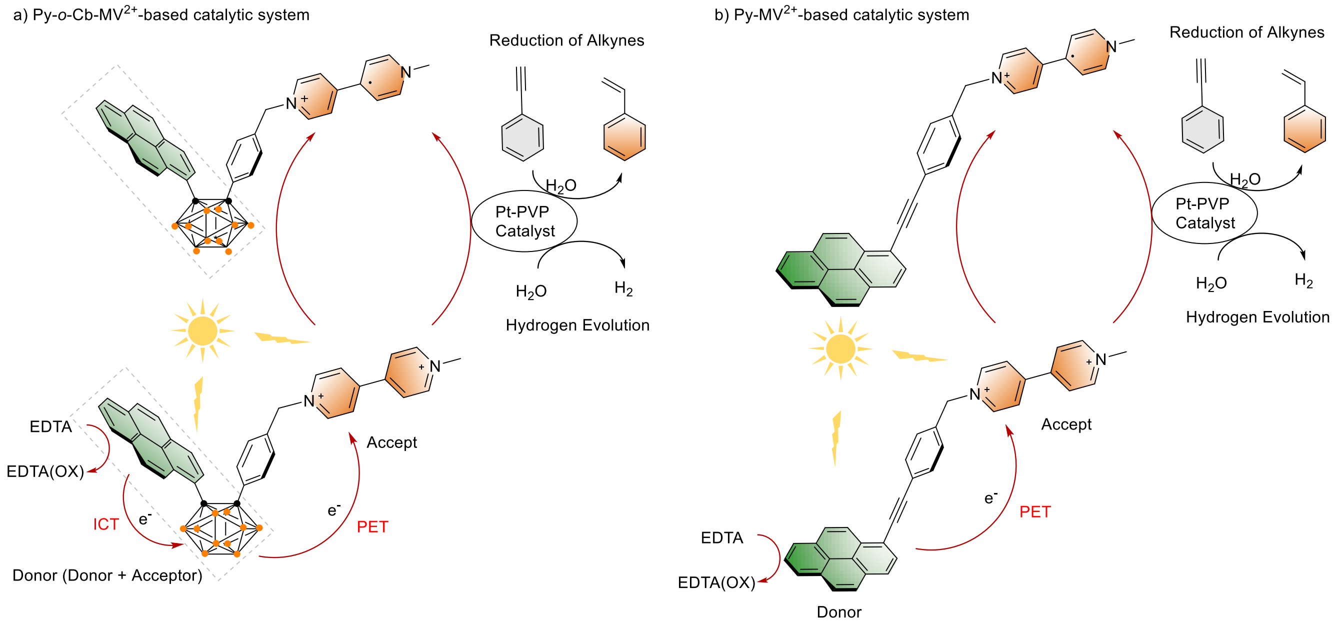

Figure S19. ICT and PET regulated photocatalytic reaction system.

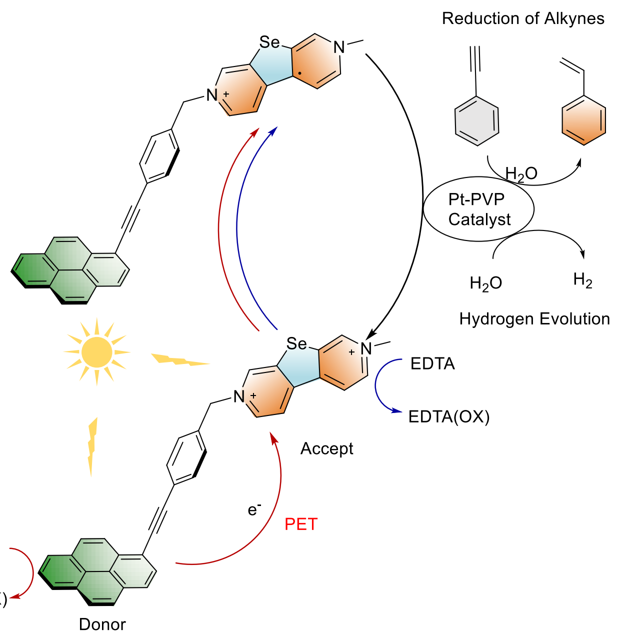

Figure S20. PET regulated photocatalytic reaction system based on **Py-SeV^{2+}**.

13. Computed UV-Vis spectra

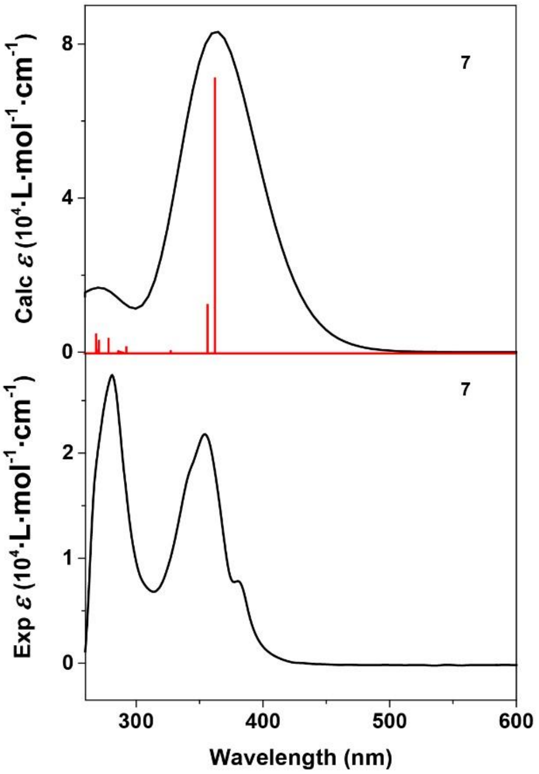

Figure S21. Computed at the PCM(THF)// TD-PBE0/6-311G(d,p)+LANL08d // PBE0/6-311G(d,p)+LANL08d level of theory , and experimental UV-vis spectra of **7**.

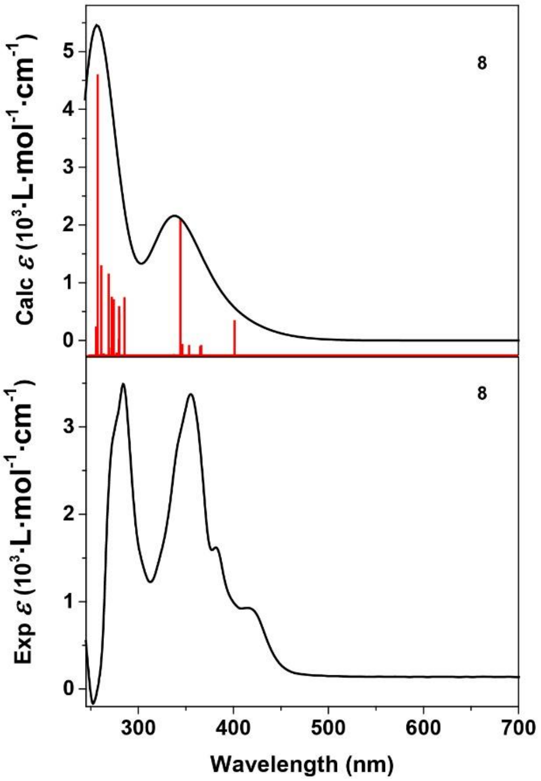

Figure S22. Computed at the PCM(THF)// TD-PBE0/6-311G(d,p)+LANL08d // PBE0/6-311G(d,p)+LANL08d level of theory , and experimental UV-vis spectra of **8**.

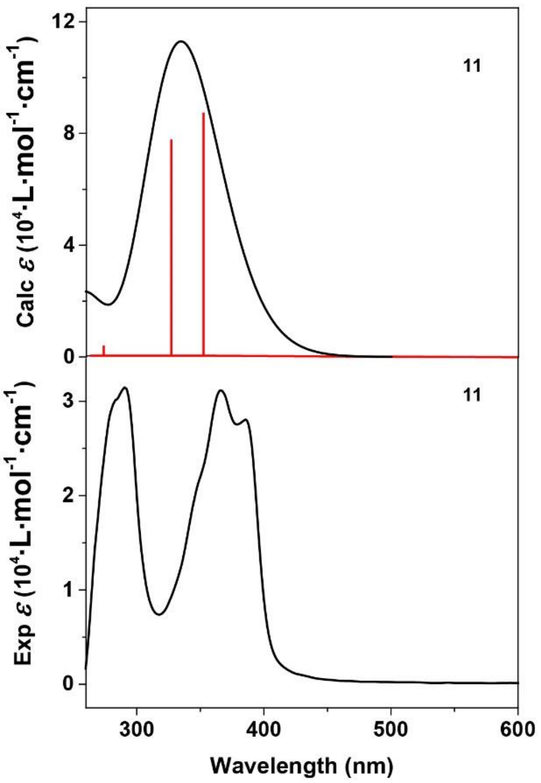

Figure S23. Computed at the PCM(THF)// TD-PBE0/6-311G(d,p)+LANL08d // PBE0/6-311G(d,p)+LANL08d level of theory , and experimental UV-vis spectra of **11**.

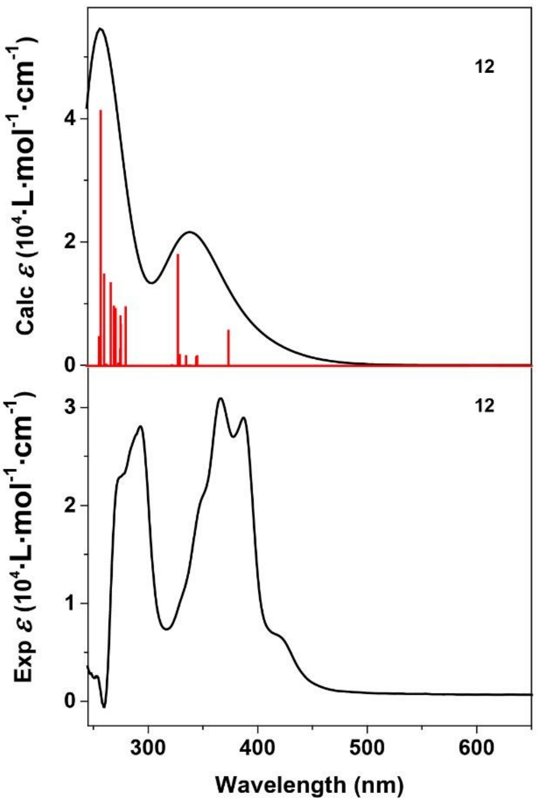

Figure S24. Computed at the PCM(THF)// TD-PBE0/6-311G(d,p)+LANL08d // PBE0/6-311G(d,p)+LANL08d level of theory , and experimental UV-vis spectra of **12**.

14. DFT Calculations

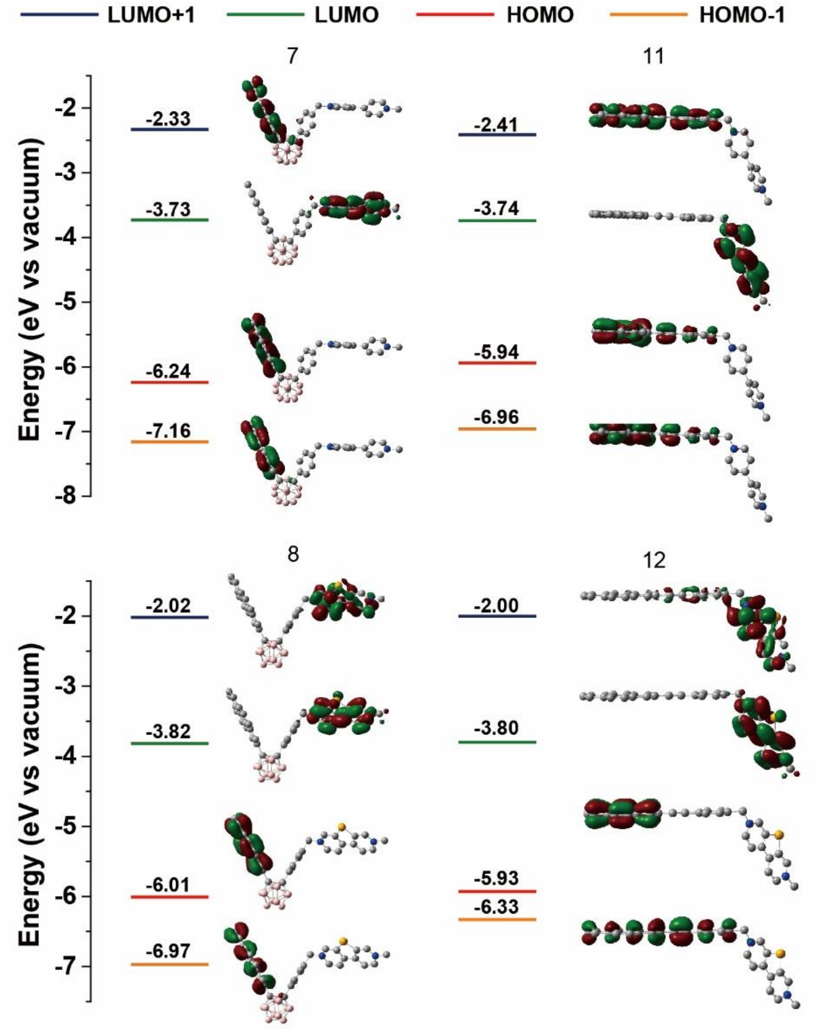

Figure S25. HOMO-1, HOMO, LUMO, and LUMO+1 energy levels of **7, 8. 11, 12** (isosurface values ±0.03).

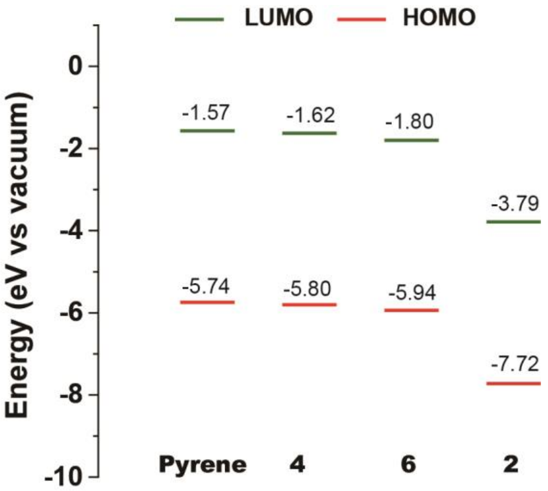

Figure S26. HOMO and LUMO energy levels of **pyrene**, **2**, **4**, **6**.

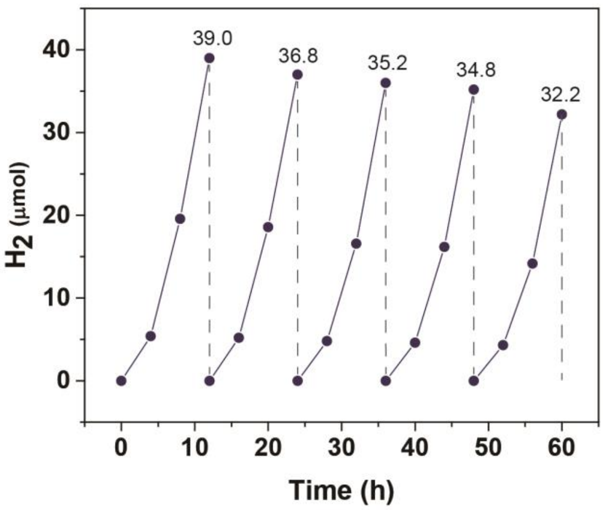

Figure S27. Hydrogen generation of **8** system upon five cycles (every twelve hours is a cycle)

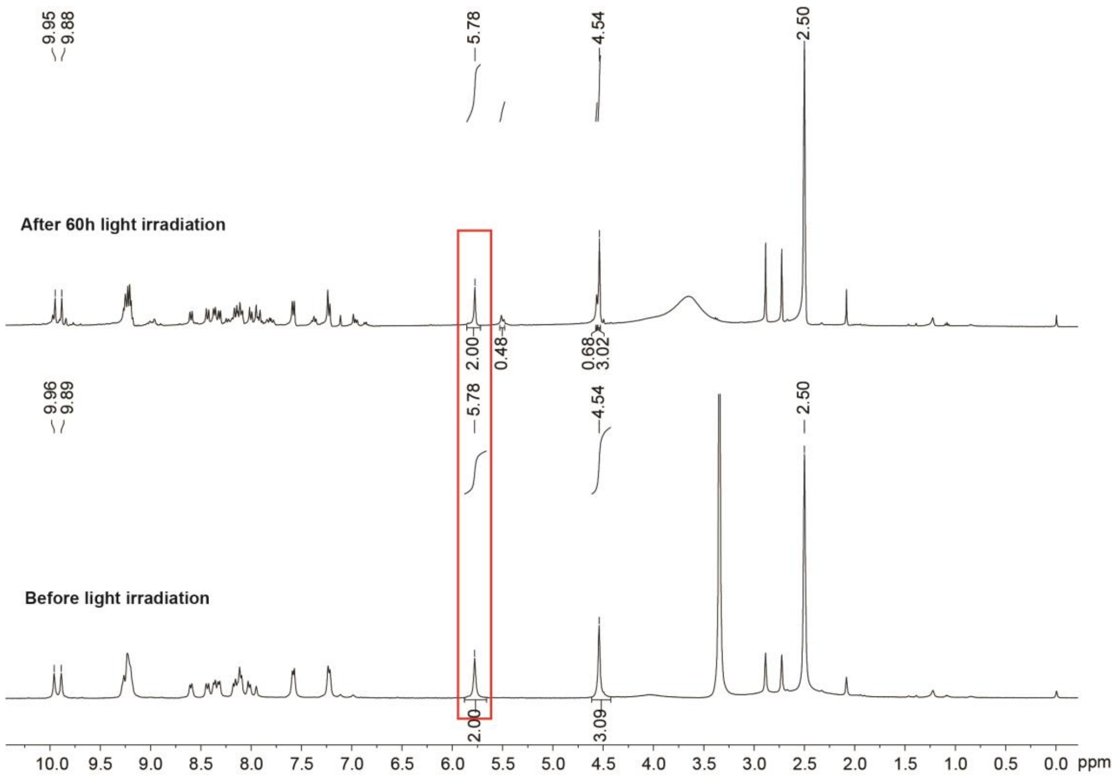

Figure S28. The ^{1}H NMR data of molecule **8** before and after 60 h light irradiation.

15. ^{1}H, ^{13}C NMR spectra

^{1}H NMR ($CDCl_3$, 400 MHz) and ^{13}C NMR ($CDCl_3$, 100 MHz) spectra of **4**

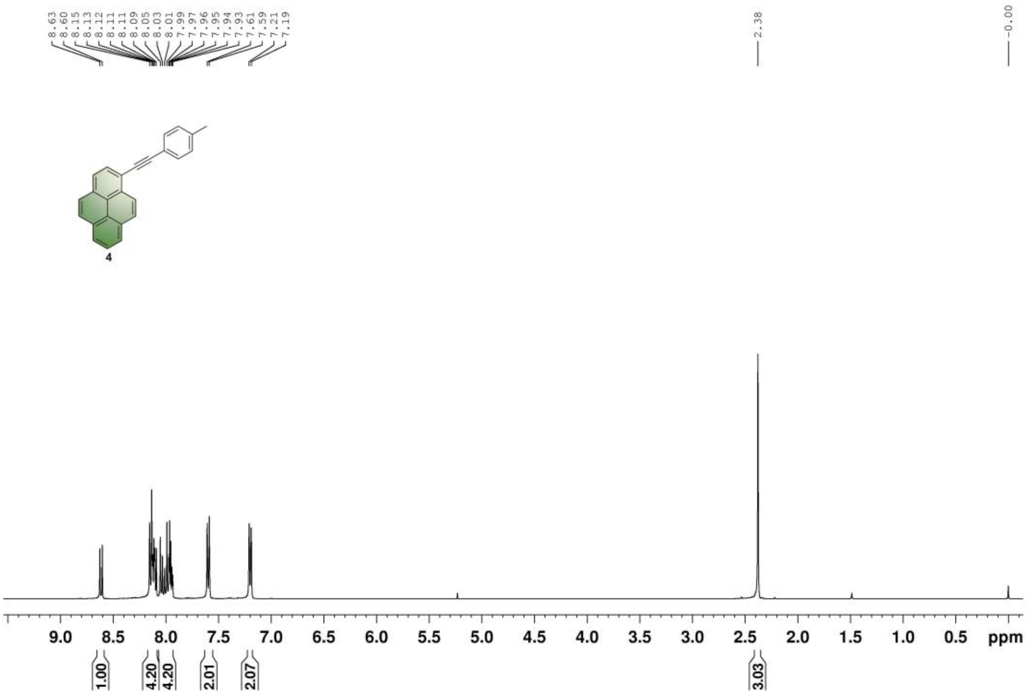

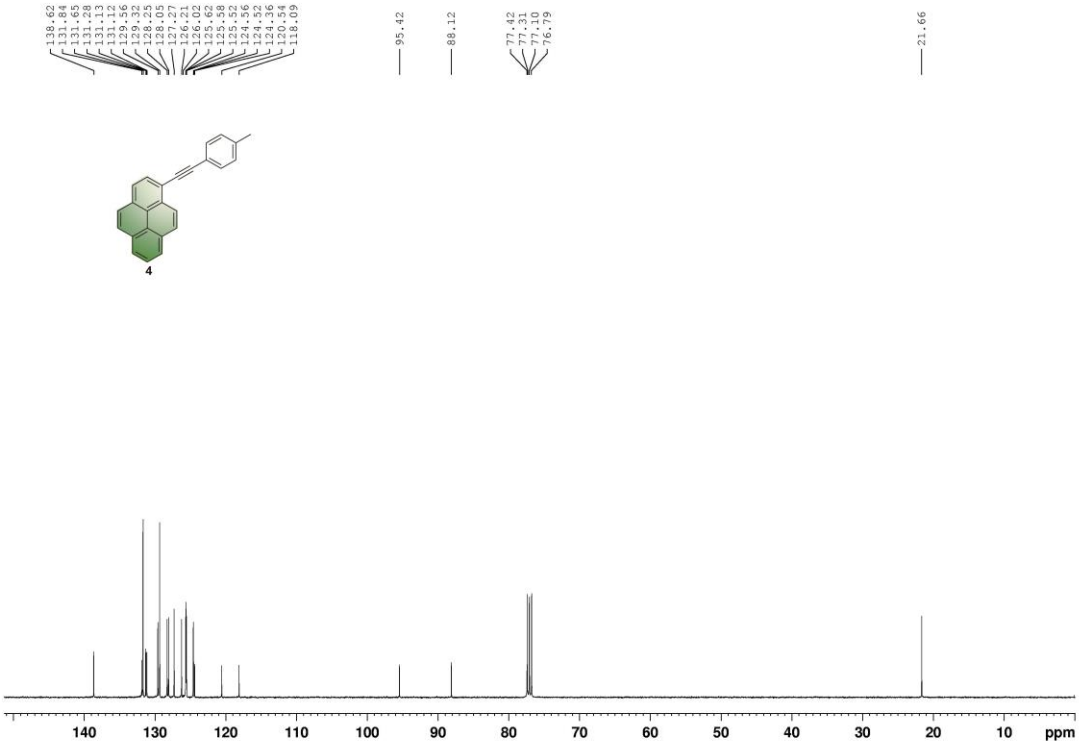

^{1}H NMR ($CDCl_3$, 400 MHz) and ^{13}C NMR ($CDCl_3$, 100 MHz) spectra of **5**

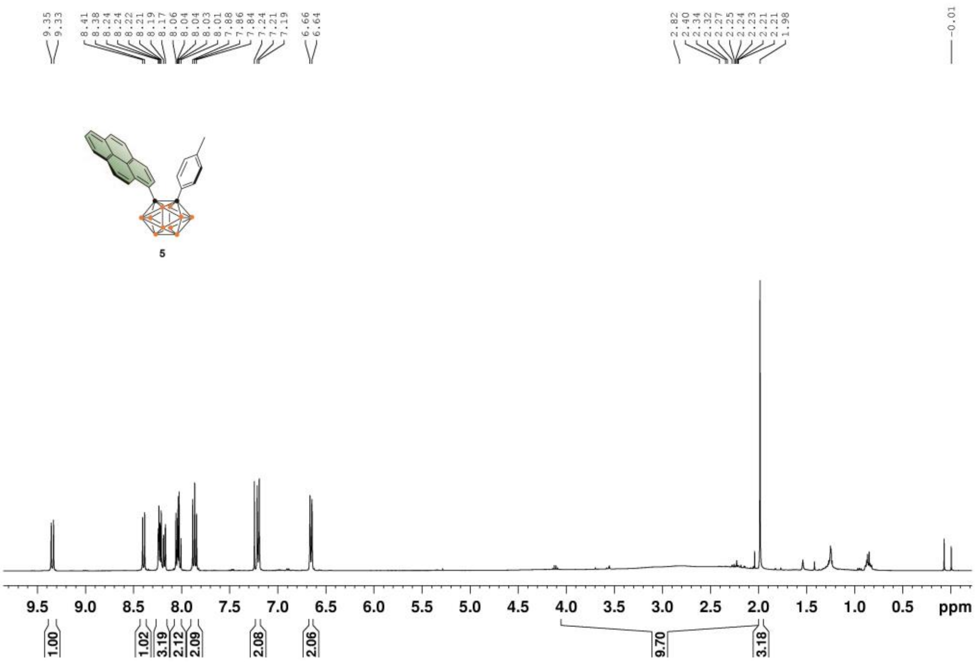

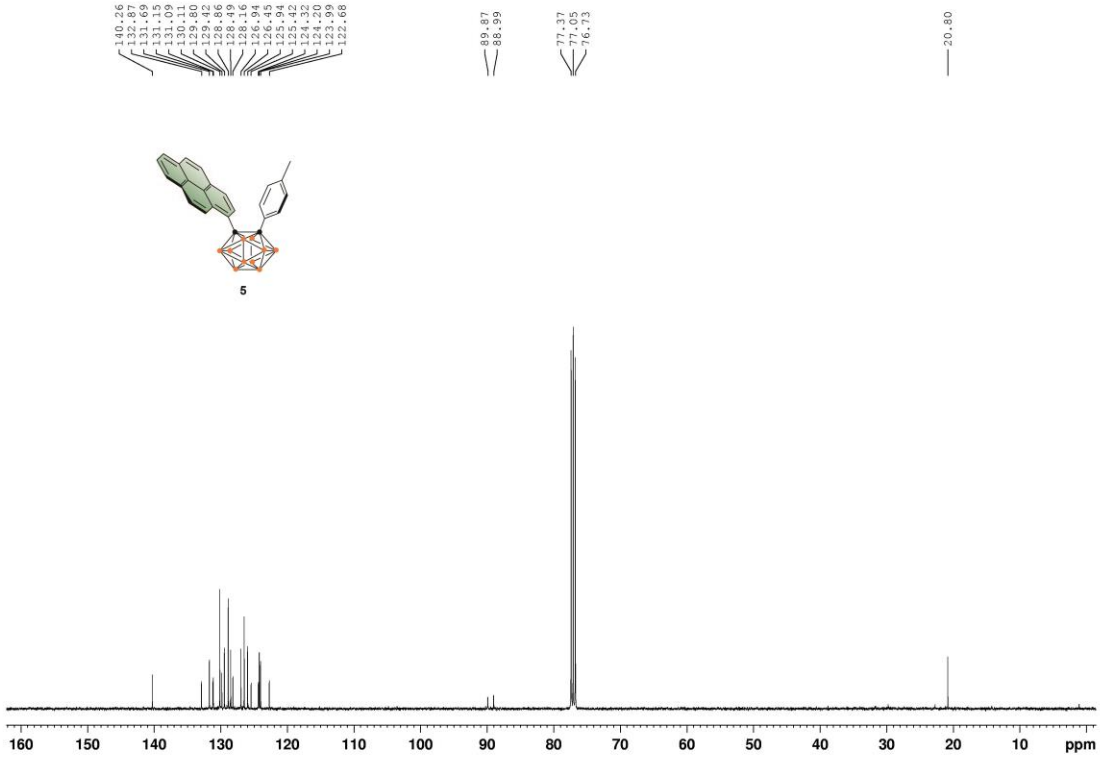

^{1}H NMR (DMSO, 400 MHz) and ^{13}C NMR (DMSO, 100 MHz) spectra of **7**

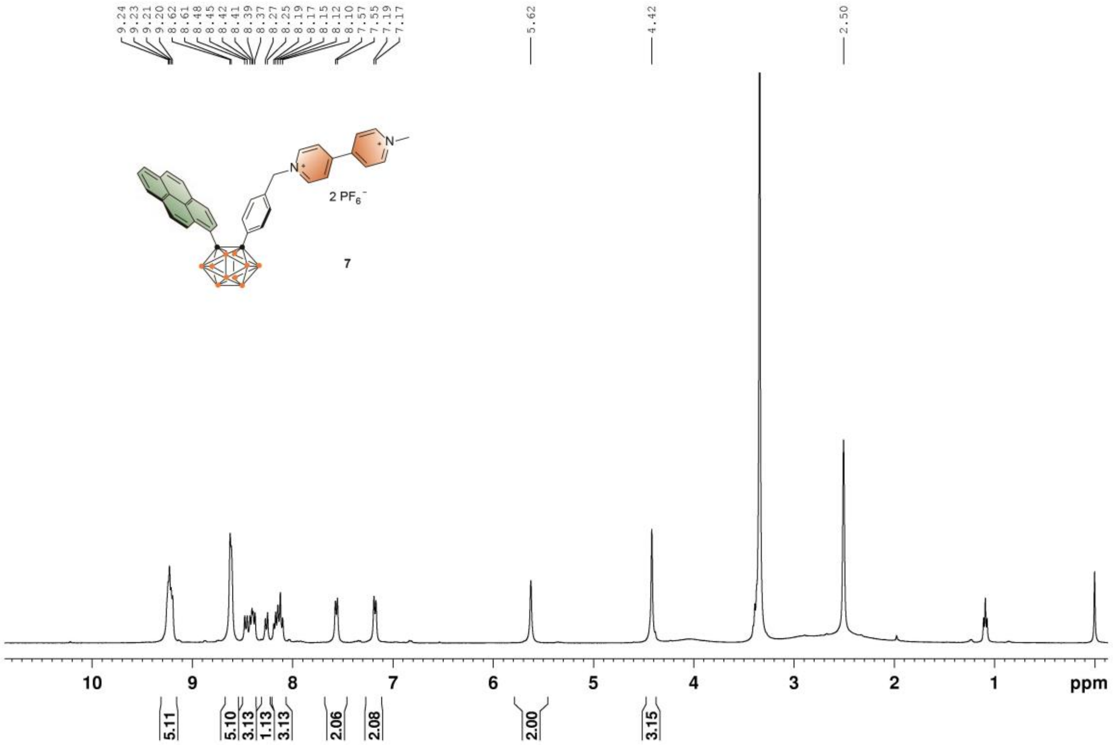

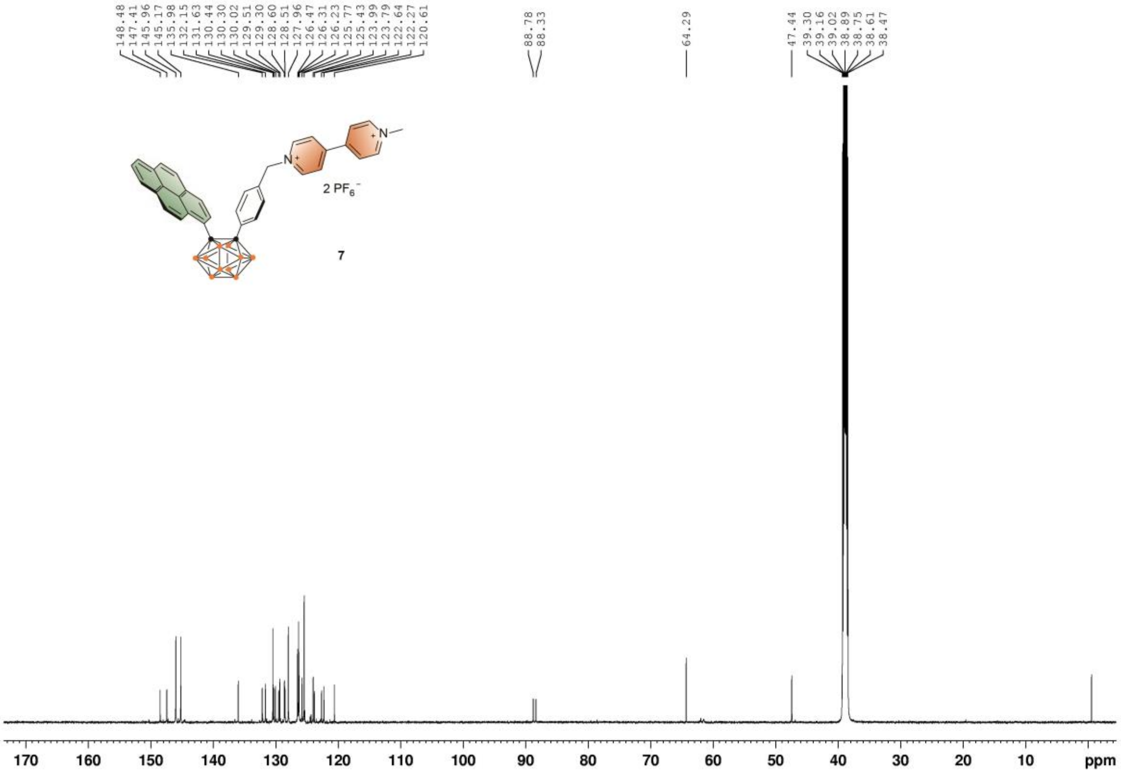

^{1}H NMR (DMSO, 400 MHz) and ^{13}C NMR (DMSO, 100 MHz) spectra of **8**

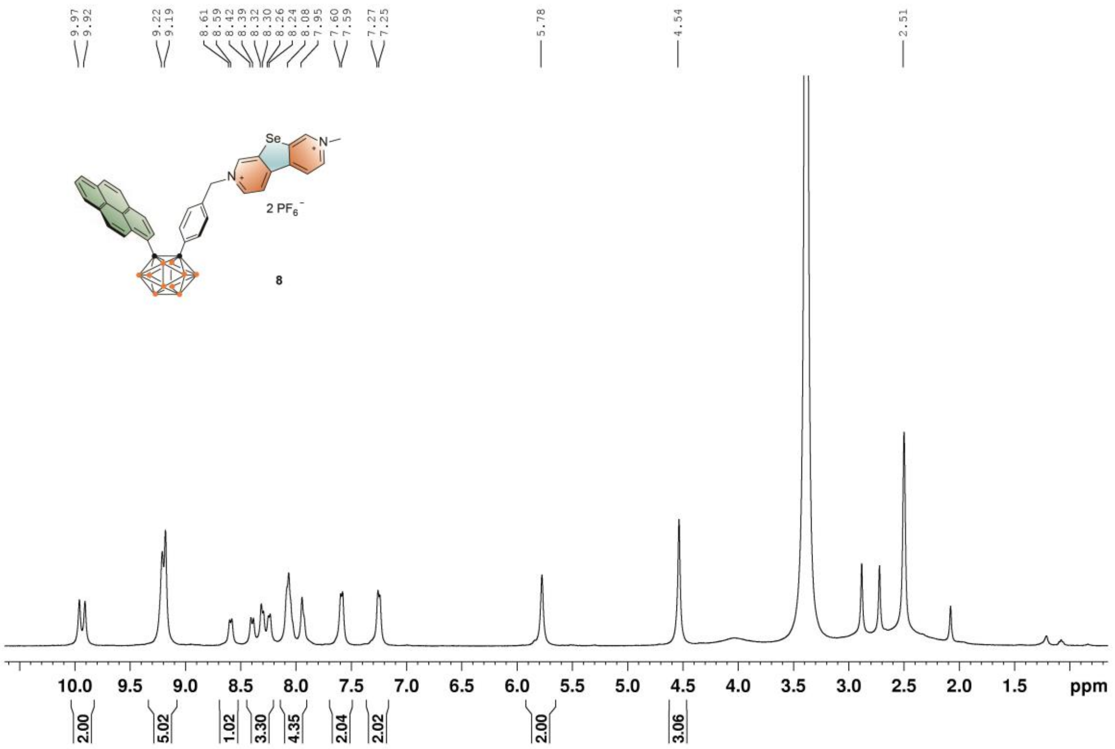

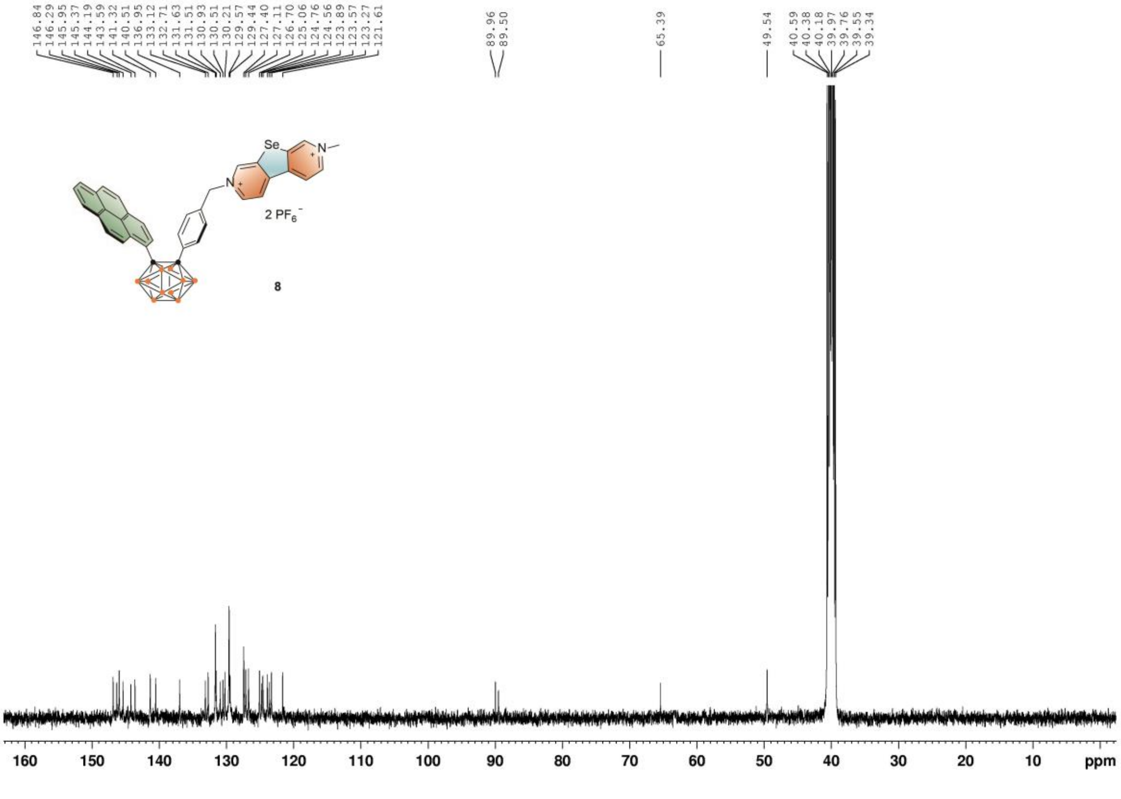

^{1}H NMR ($CDCl_3$, 400 MHz) and ^{13}C NMR ($CDCl_3$, 100 MHz) spectra of **9**

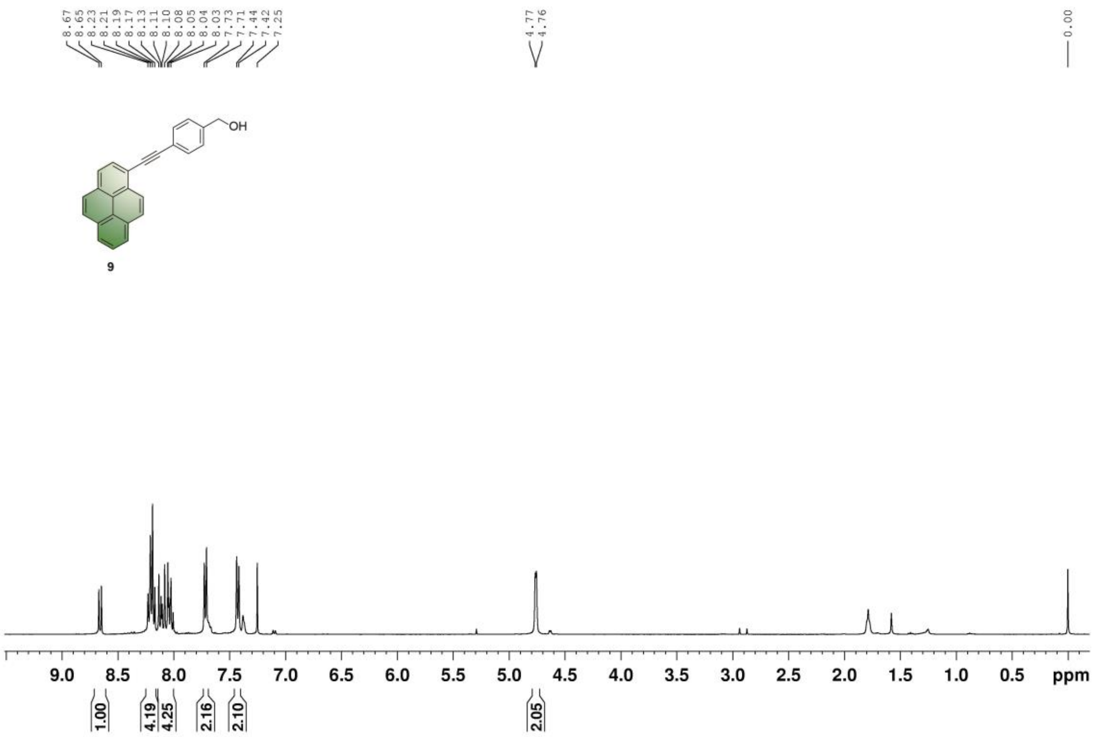

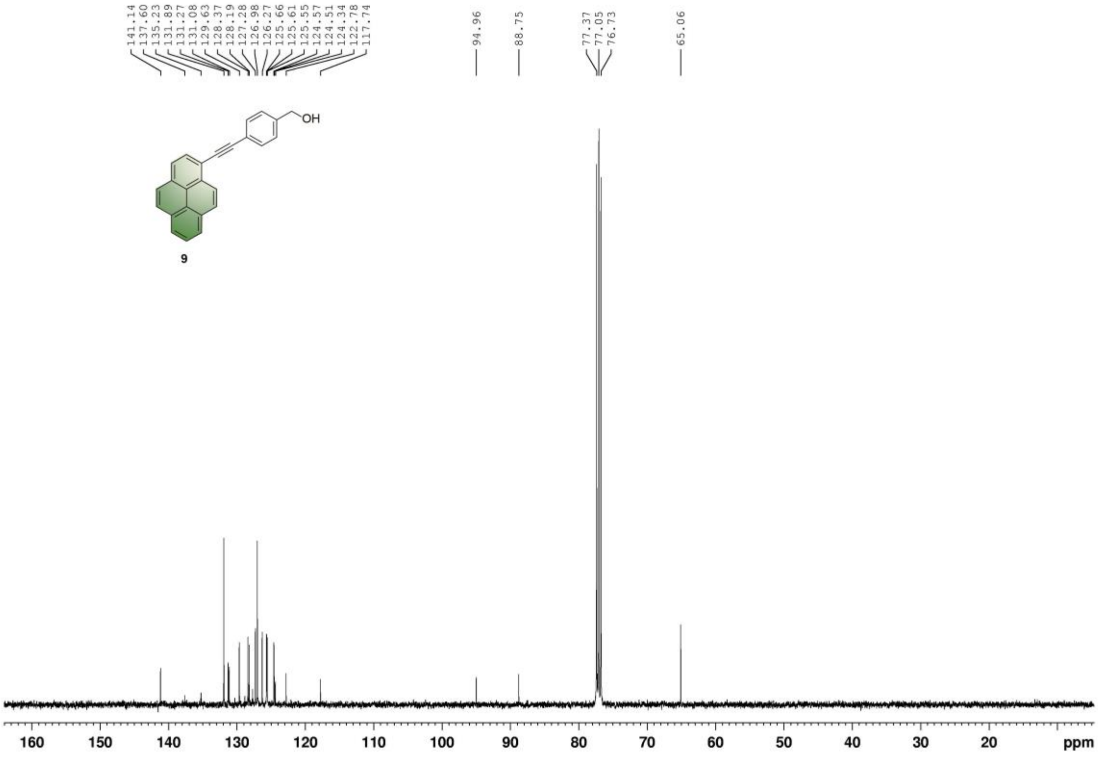

^{1}H NMR (DMSO, 400 MHz) and ^{13}C NMR (DMSO, 100 MHz) spectra of **10**

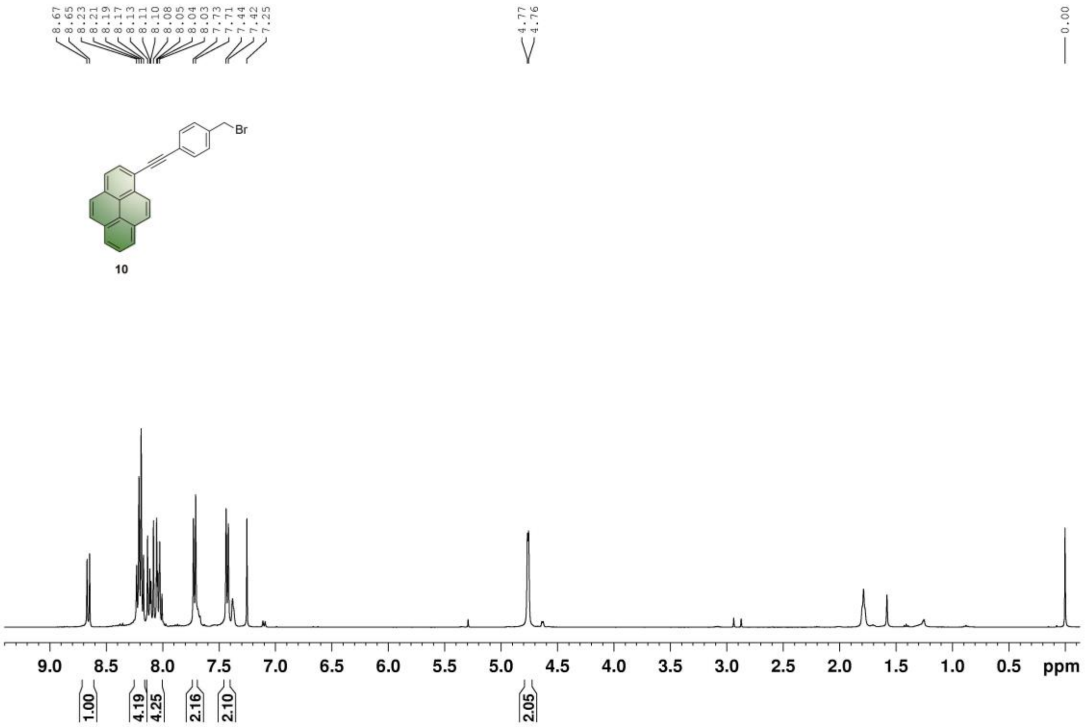

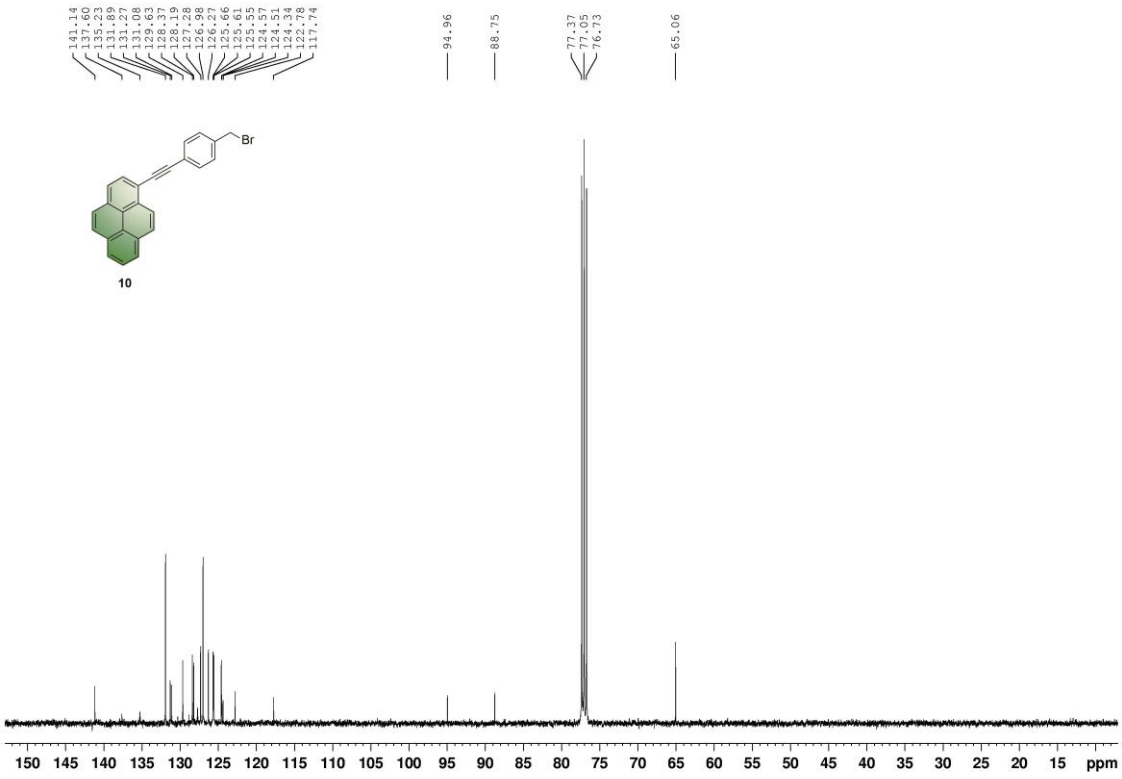
141.14
137.60
135.23
131.89
131.27
131.08
129.63
128.37
128.19
127.28
126.98
126.27
125.66
125.61
125.55
124.57
124.51
124.34
122.78
117.74
94.96
88.75
77.37
77.05
76.73
65.06
Br
10
150 145 140 135 130 125 120 115 110 105 100 95 90 85 80 75 70 65 60 55 50 45 40 35 30 25 20 15 ppm

^{1}H NMR (DMSO, 400 MHz) and ^{13}C NMR (DMSO, 100 MHz) spectra of **11**

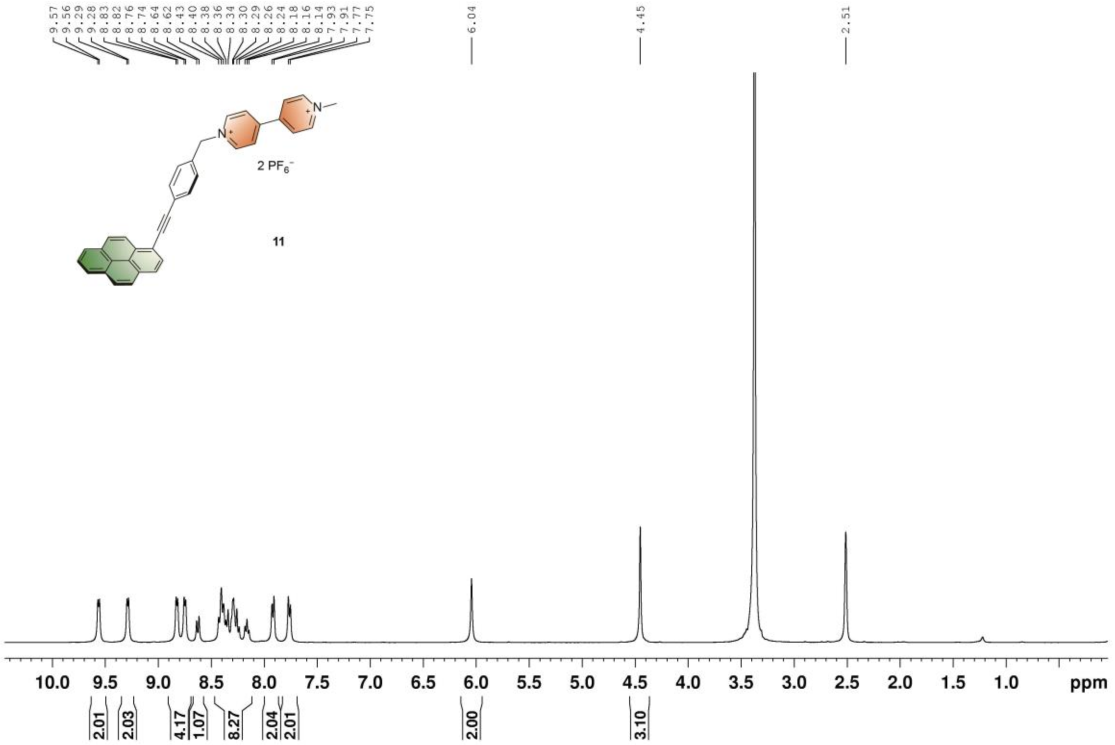

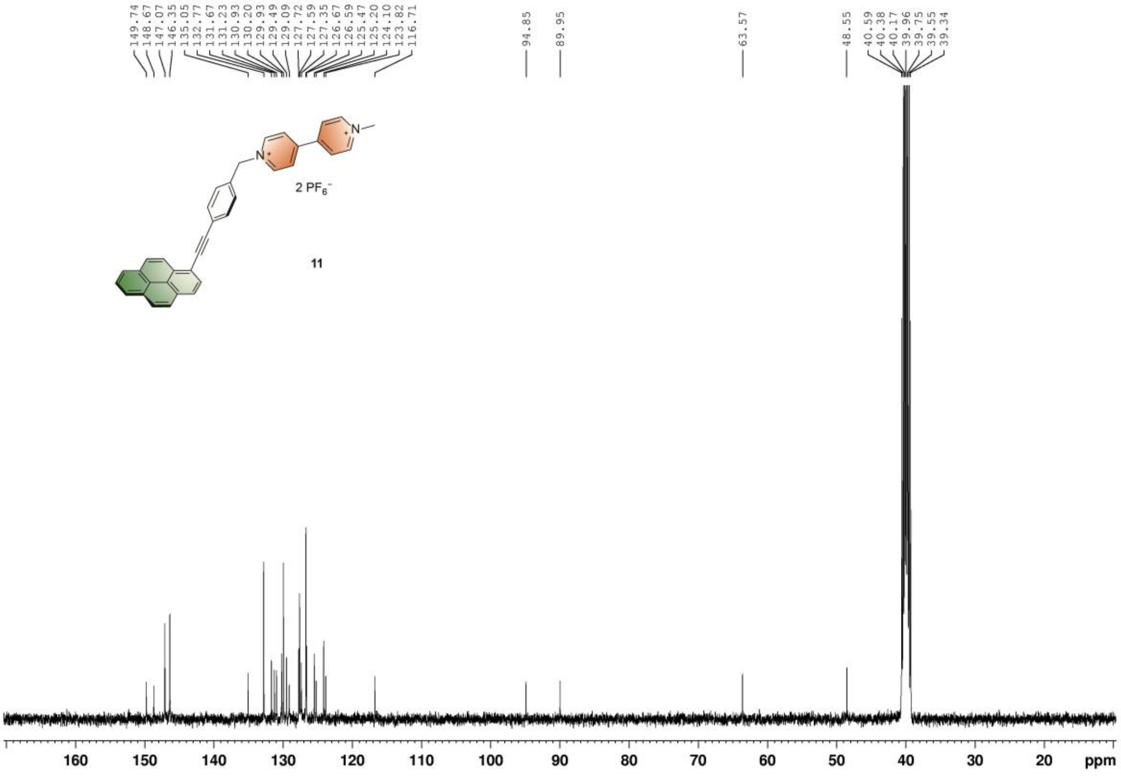

^{1}H NMR (DMSO, 400 MHz) and ^{13}C NMR (DMSO, 100 MHz) spectra of **12**

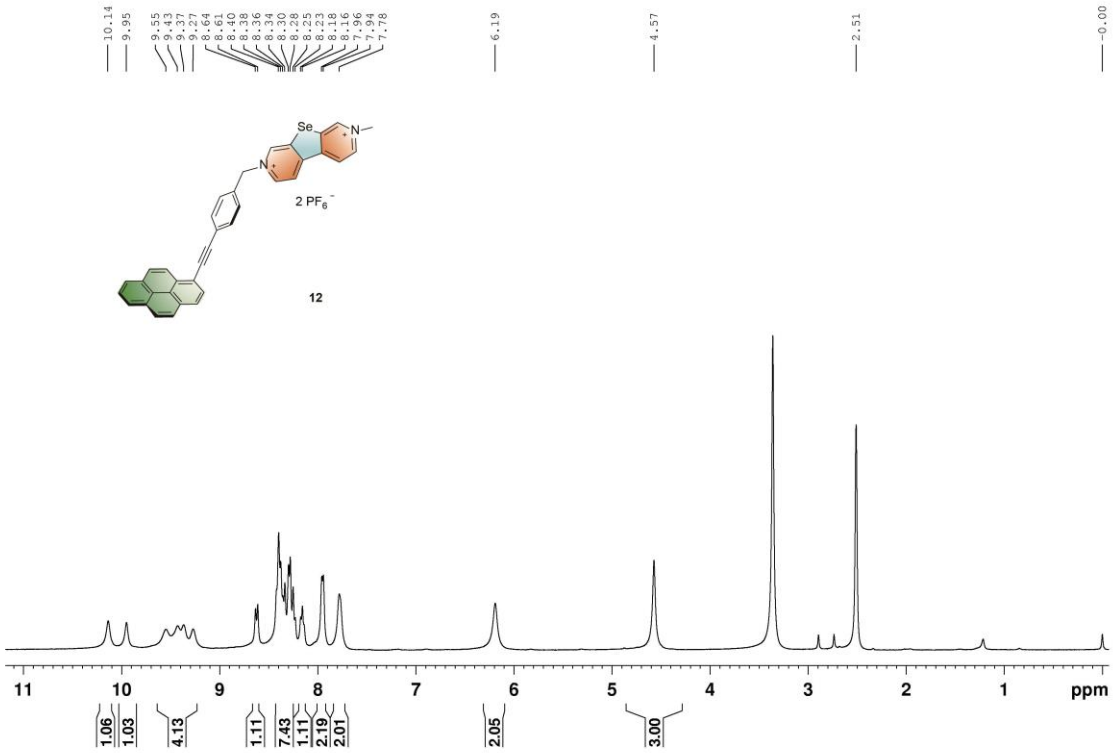

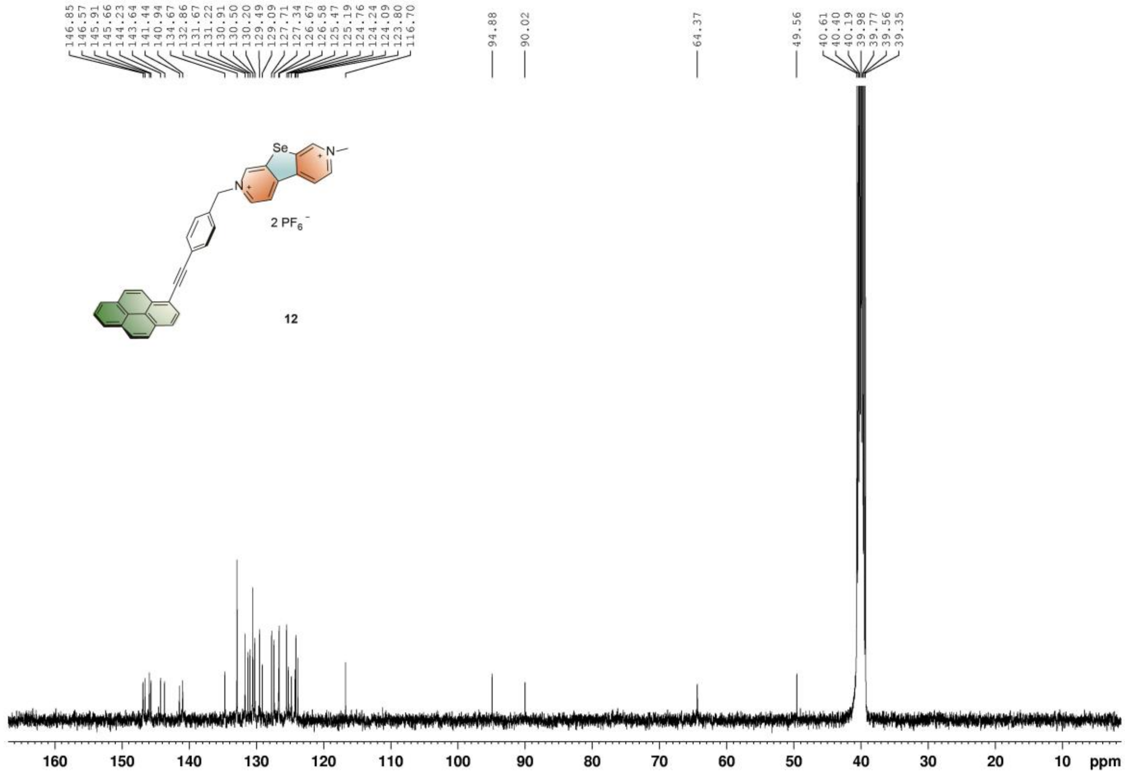

Reference

[1] K. Nishino, H. Yamamoto, K. Tanaka, Y. Chujo, *Org. Lett.* **2016**, *18*, 4064.

[2] Gaussian 09, Revision E.01, J. Frisch, G. W. Trucks, H. B. Schlegel, G. E. Scuseria, M. A. Robb, J. R. Cheeseman, G. Scalmani, V. Barone, B. Mennucci, G. A. Petersson, H. Nakatsuji, M. Caricato, X. Li, H. P. Hratchian, A. F. Izmaylov, J. Bloino, G. Zheng, J. L. Sonnenberg, M. Hada, M. Ehara, K. Toyota, R. Fukuda, J. Hasegawa, M. Ishida, T. Nakajima, Y. Honda, O. Kitao, H. Nakai, T. Vreven, J. A. Montgomery, Jr., J. E. Peralta, F. Ogliaro, M. Bearpark, J. J. Heyd, E. Brothers, K. N. Kudin, V. N. Staroverov, T. Keith, R. Kobayashi, J. Normand, K. Raghavachari, A. Rendell, J. C. Burant, S. S. Iyengar, J. Tomasi, M. Cossi, N. Rega, J. M. Millam, M. Klene, J. E. Knox, J. B. Cross, V. Bakken, C. Adamo, J. Jaramillo, R. Gomperts, R. E. Stratmann, O. Yazyev, A. J. Austin, R. Cammi, C. Pomelli, J. W. Ochterski, R. L. Martin, K. Morokuma, V. G. Zakrzewski, G. A. Voth, P. Salvador, J. J. Dannenberg, S. Dapprich, A. D. Daniels, O. Farkas, J. B. Foresman, J. V. Ortiz, J. Cioslowski, and D. J. Fox, Gaussian, Inc., Wallingford CT, **2013**.

[3] M. J. Frisch, J. E. Del Bene, *Int. J. Quantum Chem.* **2009**, *36*, 363.

[4] M. Hariharan, J. Joseph, D. Ramaiah, *J. Phys. Chem. B* **2006**, *110*, 24678.

[5] M. Stolar, J. Borau-Garcia, M. Toonen, T. Baumgartner, *J. Am. Chem. Soc.* **2015**, *137*, 3366.

Author Contributions

Xiaodong Yang, Guoping Li and Gang He conceived the idea for the study. Xiaodong Yang prepared the samples and conducted characterizations. Bingjie Zhang, Yujing Gao, Chenjing Liu, Guoping Li helped to prepare and characterize the samples. Xiaodong Yang and Gang He contributed to the DFT calculations. Bin Rao, Dake Chu, Ni Yan and Mingming Zhang

discussed the manuscript. Xiaodong Yang, Guoping Li and Gang He wrote the manuscript and all the authors revised and polished the manuscript.
